# Identification of an allosteric binding site for RORγt inhibition

**DOI:** 10.1038/ncomms9833

**Published:** 2015-12-07

**Authors:** Marcel Scheepstra, Seppe Leysen, Geert C. van Almen, J. Richard Miller, Jennifer Piesvaux, Victoria Kutilek, Hans van Eenennaam, Hongjun Zhang, Kenneth Barr, Sunil Nagpal, Stephen M. Soisson, Maria Kornienko, Kristen Wiley, Nathaniel Elsen, Sujata Sharma, Craig C. Correll, B. Wesley Trotter, Mario van der Stelt, Arthur Oubrie, Christian Ottmann, Gopal Parthasarathy, Luc Brunsveld

**Affiliations:** 1 Laboratory of Chemical Biology, Department of Biomedical Engineering and Institute of Complex Molecular Systems, Eindhoven University of Technology, PO Box 513, Eindhoven 5600MB, The Netherlands; 2 Merck Research Laboratories, 33 Avenue Louis Pasteur, Boston, Massachusetts 02115, USA; 3 Merck Research Laboratories, Molenstraat 110, Oss 5342 CC, The Netherlands; 4 Merck Research Laboratories, 770 Sumneytown Pike, West Point, Pennsylvania 19486, USA; 5 Merck Research Laboratories, 503 Louise Lane, North Wales, Pennsylvania 19454, USA

## Abstract

RORγt is critical for the differentiation and proliferation of Th17 cells associated with several chronic autoimmune diseases. We report the discovery of a novel allosteric binding site on the nuclear receptor RORγt. Co-crystallization of the ligand binding domain (LBD) of RORγt with a series of small-molecule antagonists demonstrates occupancy of a previously unreported allosteric binding pocket. Binding at this non-canonical site induces an unprecedented conformational reorientation of helix 12 in the RORγt LBD, which blocks cofactor binding. The functional consequence of this allosteric ligand-mediated conformation is inhibition of function as evidenced by both biochemical and cellular studies. RORγt function is thus antagonized in a manner molecularly distinct from that of previously described orthosteric RORγt ligands. This brings forward an approach to target RORγt for the treatment of Th17-mediated autoimmune diseases. The elucidation of an unprecedented modality of pharmacological antagonism establishes a mechanism for modulation of nuclear receptors.

## Abstract (web summary)

Upon the binding of small ligands, nuclear receptors regulate the transcription of genes that are associated with a number of disease mechanisms. Here, the authors report on a novel allosteric ligand binding site on the nuclear receptor RORγt.

## Graphical abstract

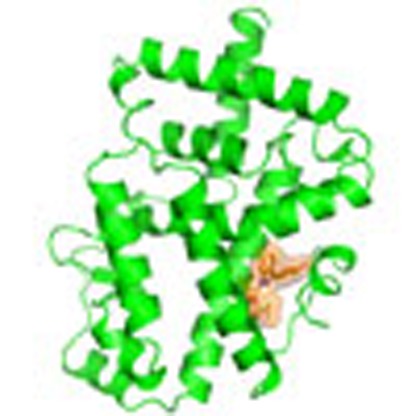

Nuclear receptors (NRs) modulate transcription of particular sets of genes on binding of small lipophilic ligands and thereby regulate physiological parameters of cellular function^1^. NRs are also important pathological regulators in diseases such as cancer, diabetes and autoimmune disorders. This combination of characteristics of NRs has given rise to some of the most notable pharmaceutical agents of the past century^2^. The retinoic-acid-receptor-related orphan receptor (ROR) is a NR subclass that demonstrates great therapeutic potential^3^. In particular, RORγt, whose activity is required for the proliferation and functionality of immune Th17 cells, is the subject of intense investigation to modulate its activity to achieve clinical benefit^4^,^5^,^6^. Th17 cells exert an inflammatory, pathological role in autoimmune diseases^7^,^8^ and on stimulation produce pro-inflammatory cytokines^9^. Antibodies directed against the cytokine IL17 have been clinically successful, proving the potential of targeting the Th17/IL17 axis^10^. Active RORγt is a prerequisite for the differentiation of T cells into Th17 cells^11^,^12^. Small-molecule inhibition of RORγt has therefore been brought forward as a novel strategy for the treatment of autoimmune diseases^13^,^14^.

NRs are characterized by the ability to bind small ligands at a highly conserved hydrophobic orthosteric-binding pocket located within the protein's ligand-binding domain (LBD)^1^. A typical NR LBD exhibits a three-layered fold of ∼12 alpha helices and 2–3 β-strands. Ligand binding in this pocket can activate or inhibit the receptor to various degrees^15^. Helix 12 (H12, also called activation function-2, AF-2) can adopt distinct conformations in response to ligand binding, regulating the interaction of the LBD with cofactor proteins with resulting changes in gene transcription at a particular locus. Typically, on the binding of an agonist, H12 is stabilized in a conformation that facilitates the binding of a coactivator^16^,^17^. Conversely, antagonist binding induces a different H12 conformation unsuitable for coactivator binding. NR drugs thus bind to this orthosteric-binding pocket and act as molecular ‘switches' that control NR transcriptional activity due to the positioning of H12 (ref. 18). This canonical ligand binding is associated with selectivity issues and mutation-induced antagonist/agonist switches for different NRs and therefore molecules that occupy allosteric-binding sites on NRs are highly sought after^19^,^20^,^21^. Such allosteric modulation might be expected to induce conformational effects that are not dependent on competition with endogenous ligands and could provide enhanced potency/efficacy or greater specificity over canonical ligands.

We previously identified a novel series of RORγt inhibitors^22^. Here we characterize the mode of action of these inhibitors to guide an optimization program and surprisingly find a novel binding mode, thereby identifying the first allosteric-binding pocket for a highly potent, cellular active small NR ligand. Structural, biochemical and cellular data reveal that the unprecedented allosteric-binding modality confers both high potency and selectivity to RORγt for these novel antagonists.

## Results

### Helix 12 repositions to generate a novel binding pocket

**Figure 1:**
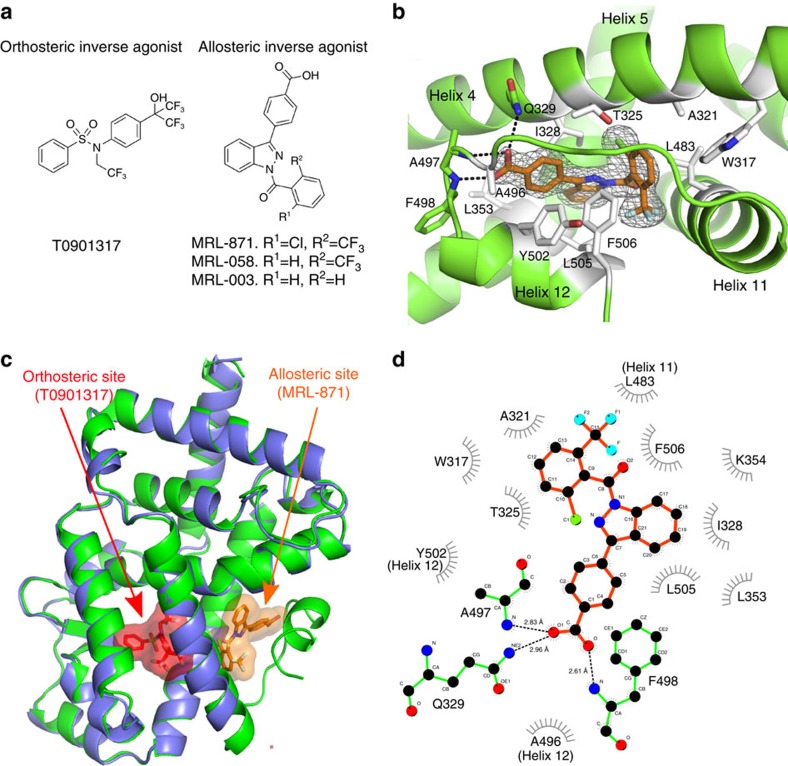
Co-crystal structure of RORγt with MRL-871. (**a**) Chemical structures of a representative orthosteric ligand T0901317 (ref. 27), binding to the canonical site of NRs, and indazoles **MRL-871**, **MRL-058** and **MRL-003**. (**b**) Zoomed-in view of **MRL-871** in the novel allosteric-binding pocket of RORγt formed by helices 4, 5, 11 and 12. **MRL-871** shown as orange sticks in the electron density. The RORγt residues involved in hydrophobic interactions and hydrogen bonding are shown as white and green sticks, respectively. (**c**) Superposition of RORγt (blue) in complex with orthosteric ligand T0901317 shown in red (PDB ID 4NB6)^26^ and RORγt (green) in complex with **MRL-871**, shown in orange. **MRL-871** (orange) sits in a new allosteric pocket in direct contact with H12 inducing its repositioning. Orthosteric ligand T0901317 destabilizes H12, which is therefore not visible in the blue structure. (**d**) A two-dimensional plot showing the interactions between **MRL-871** and the surrounding amino acids.

Literature suggests that the RORs feature ligand-independent transcription, with their LBDs partially in a conformation promoting coactivator binding^23^. Biological data and the co-crystal structures of RORγt LBD bound to hydroxycholesterols^24^, and synthetic inverse agonistic ligands such as T0901317 (Fig. 1a) have shown that the RORγt LBD is still structurally responsive to ligands. For RORγt, reports suggest that multiple small molecules affect antagonism via binding to the canonical orthosteric site^13^. A high-throughput screen for molecules that disrupt the interaction of the RORγt-LBD with steroid receptor coactivator-1 (SRC-1) cofactor peptide, followed by a hit optimization program, led to the identification of indazoles as a novel class of RORγt inhibitors, typified by **MRL-871** (Fig. 1a)^22^. To elucidate the molecular basis of RORγt modulation by **MRL-871**, we performed co-crystallization studies with an equimolar complex of RORγt-LBD and **MRL-871**. Co-crystals grew in two different space groups (Table 1). Crystals in space group R32:H had unit cell dimensions of *a*=*b*=173.8, *c*=67.2 Å, diffracted to 2.3 Å resolution and contain one molecule per asymmetric unit. Crystals in space group P6122 had unit cell dimensions of *a*=*b*=108.5, *c*=104.7 Å, diffracted to 2.2 Å resolution and also contain one molecule per asymmetric unit. The experimental electron density maps from both conditions showed clear density for all features of the protein, including **MRL-871**. The overall structures from both sets were identical in all the features and the ligand conformation. The RORγt LBD crystallized with the typical NR arrangement of helices 1–11, but with the H12 positioned in a conformation unprecedented for all NR LBD complexes reported to date (Fig. 1c, green). On top of that, the crystal structure revealed the binding site of **MRL-871** to be different from the canonical orthosteric NR ligand-binding site. **MRL-871** binds instead to a previously unidentified allosteric pocket in the RORγt LBD, located distal to the classical binding site (Fig. 1c, orange). Crystal structure analysis shows that this allosteric pocket, absent in the classical NR-folding motif, is formed by helices 4, 5, 11 and the reoriented flexible H12 (Fig. 1b). The ortho-substituted trifluoromethyl and chloro moieties are spatially clearly positioned and impart a specific rotation to the phenyl group of **MRL-871** and address hydrophobic sites in the allosteric pocket (Fig. 1b,d). Hydrogen-bonding interactions exist between the molecule's carboxylic acid group and the side chain of RORγt residue Q329 as well as the main-chain amide hydrogen atoms of residues A497 and F498. Apart from these distinct polar interactions, the newly generated allosteric pocket is predominantly hydrophobic, because of the amino-acid side chains of residues on helices 4, 5, 11 and, notably, H12 and the activation function loop between helices 11 and 12 (AF-2 domain). The arrangement of the RORγt LBD in the presence of **MRL-871** generates a druggable molecular-binding site critically regulated by favourable interactions of **MRL-871** with the AF-2 domain. These interactions reposition the highly flexible H12 in a conformation unique among reported NR ligand structures, of either the agonist or antagonist type, and distinct from the previously reported RORγt agonist-bound state^1^,^6^,^13^,^14^. The unique conformation induced by the binding of **MRL-871** prevents interaction with cofactor peptides, which typically bind RORγt through a conserved LXXLL motif, at the AF-2.

### Functional RORγt inhibition via allosteric inverse agonism

**Figure 2:**
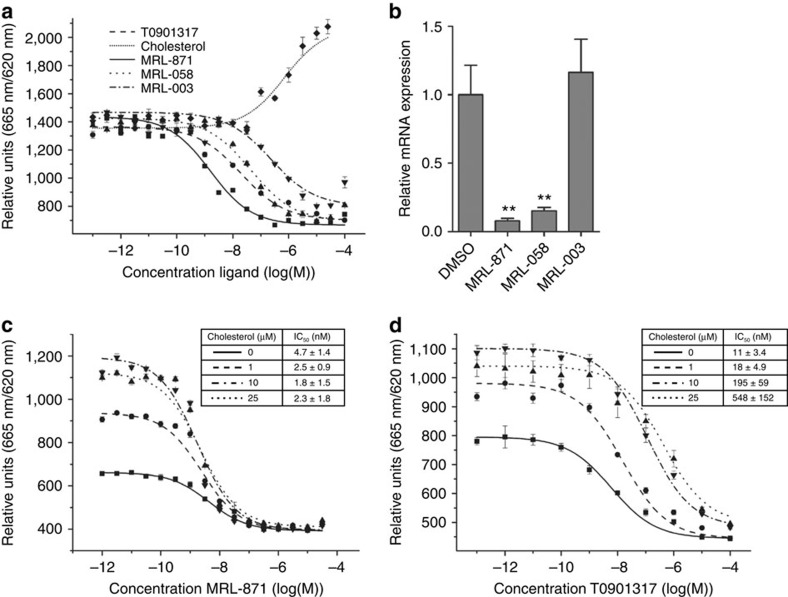
Allosteric inhibition. (**a**) TR-FRET assay showing the effect of the agonist cholesterol, the inverse agonist T0901317 and indazoles **MRL-871**, **MRL-058** and **MRL-003** on cofactor recruitment to the RORγt LBD in a dose-dependent manner. **MRL-871**, **MRL-058**, **MRL-003** and T0901317 function as inhibitors, while cholesterol promotes cofactor binding to RORγt. Error bars are defined as s.d. (*n*=3). (**b**) IL17a mRNA expression in EL4 cells treated with **MRL-871**, **MRL-058**, **MRL-003** (10 μM, 24 h) or DMSO. The level of IL17a mRNA expression was normalized to that of GAPDH expression. All data are expressed as the mean±s.e.m. (*n*=6). Statistical analysis was performed using an one-way analysis of variance comparing against the DMSO control following Dunnett *post hoc* test. Error bars denote s.e.m. **P<0.01. (**c**) Inhibition of cofactor binding by the allosteric modulator **MRL-871** is independent of cholesterol concentration (IC_50_ 4.7±1.4 nM; 2.5±0.9 nM; 1.8±1.5 nM; 2.3±1.8 nM at 0, 1, 10 and 25 μM cholesterol, respectively). Error bars are defined as s.d. (*n*=3). (**d**) Inhibition of cofactor binding by ligand T0901317 depends on cholesterol concentration (IC_50_ 11±3.4 nM; 18±4.9 nM; 195±59 nM; 548±152 nM at 0, 1, 10 and 25 μM cholesterol, respectively). Error bars are defined as s.d. (*n*=3).

To determine the functional effect of the binding of these novel modulators and the resulting RORγt conformational changes, two variants of **MRL-871** (**MRL-058** and **MRL-003**) were also prepared and tested alongside an agonist and canonical inverse agonist in an AlphaScreen cofactor peptide recruitment assay. Indazoles **MRL-871** and its derivatives **MRL-058** and **MRL-003** all inhibited coactivator binding in a dose-dependent manner (Fig. 2a) with half-maximum inhibitory concentration (IC_50_) values of 7±1 nM (**MRL-871**), 98±23 nM (**MRL-058**) and 280±117 nM (**MRL-003**), showing the antagonistic profile of the series. Cholesterol, binding as agonist to the LBD, functioned as a weak activator, further enhancing the interaction of the RORγt–LBD with a cofactor peptide^25^. The inverse agonist T0901319 demonstrated an IC_50_ value of 24±13 nM in this assay, which is in good agreement with previously reported values, but less potent than **MRL-871** (refs 26, 27). The data thus show that the allosteric-binding mode of the novel modulators as observed in the crystal structures translates into potent inhibition of coactivator peptide binding to RORγt–LBD. Furthermore, these data also indicate that both ortho-substituents on the benzamide moiety are required to enhance RORγt-binding affinity, which suggest that the observed binding mode in the crystal structure is most optimal.

To corroborate our structural findings and gain more insight in the mode of action for these compounds, competitive binding assays for both **MRL-871** (Fig. 2c) and T0901317 (Fig. 2d) were performed against fixed concentrations of cholesterol. On the basis of the structural data, we hypothesized that **MRL-871** should not compete with cholesterol for the orthosteric ligand-binding pocket, because they bind into different pockets. As such, the IC_50_ value of **MRL-871** for inhibition of the RORγt coactivator interaction should be independent of cholesterol concentration. Increasing concentrations of cholesterol increase the RORγt coactivator interaction, in line with the agonistic properties of cholesterol, as evidenced by an increased fluorescence ratiometric level with cofactor peptide (Fig. 2c,d). **MRL-871**, however, effectively antagonizes the RORγt coactivator peptide recruitment interaction, independent of cholesterol concentration. The competitive cofactor-binding assay for **MRL-871** showed no significant change in IC_50_ value when performed in the presence of different concentrations of cholesterol (Fig. 2c). In contrast, the inverse agonist T0901317 competes for the same binding site as cholesterol, resulting in a competitive displacement and a cholesterol concentration-dependent increase of the IC_50_ value for T0901317 (Fig. 2d); that is, RORγt binding and inhibition by T0901317 becomes less potent in the presence of agonistic ligands. Together, these data demonstrate that the allosteric inhibition of coactivator binding to RORγt by **MRL-871** is both potent and independent of orthosteric site occupancy.

RORγt mediates IL17a gene expression in EL4 cells^4^. The EL4 murine lymphoblast cell line constitutively expresses RORγt, which drives production of IL17a (ref. 28). To confirm that allosteric RORγt modulation has also functional relevance at the cellular level, EL4 cells were treated with 10 μM of modulators **MRL-871**, **MRL-058** and **MRL-003** for 24 h and IL17a messenger RNA (mRNA) levels were measured by quantitative reverse transcriptase PCR (RT–PCR) (Fig. 2b). Treatment of EL4 cells with the more potent **MRL-871** and **MRL-058** significantly reduced the IL17a mRNA levels, while the weaker **MRL-003** did not reduce the mRNA levels, consistent with the lower biochemical activity observed for this compound. This result thus demonstrates that functional modulation of the allosteric pocket by small molecules results in cellular responses (that is, reduced gene transcription).

### Structural basis for ligand potency

The cofactor recruitment assay described above provides an indirect assessment of modulator potency since the site of cofactor interaction does not directly overlap with the binding site of the indazoles. Therefore, an orthogonal assay that directly and selectively probes the novel allosteric RORγt-binding site is desirable for screening, characterization and optimization purposes. We used the co-crystal structure of RORγt and **MRL-871** (Fig. 1c) to rationally design a synthetic ligand analogue containing a time-resolved fluorescence resonance energy transfer (TR-FRET) acceptor. AlexaFluor 647 was connected to the six position of **MRL-871** via a short molecular spacer (Supplementary Fig. 1); this site of the molecule protrudes into an open channel in the crystal structure. This probe molecule proved to have a robust TR-FRET signal at low concentrations in the presence of His-tagged RORγt–LBD and an anti-His Europium chelate antibody. Titration studies in a TR-FRET assay revealed a *K*_d_ of 100±24 nM with the RORγt–LBD (Supplementary Fig. 1). When the parent **MRL-871** was tested for the ability to compete with the allosteric probe, the resulting IC_50_ value was similar to that observed in cofactor recruitment assays (Table 2).

**Figure 3:**
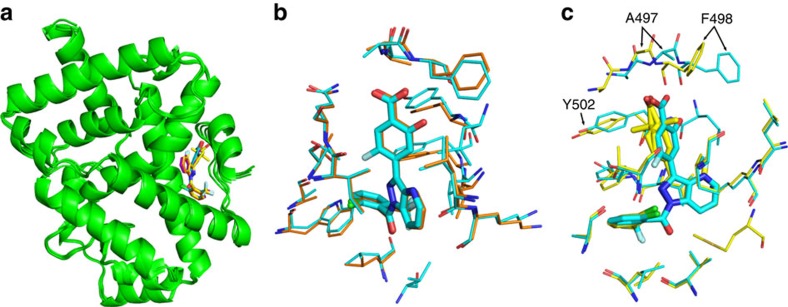
Co-crystal structure of RORγt with MRL-871, MRL-299, MRL-367 and MRL-673. (**a**) Overlay of RORγt (green) co-crystallized with **MRL-871**, **MRL-299**, **MRL-367** and **MRL-673**. For an overall structural comparison of RORγt in complex with MRL-299 and MRL-367 see Supplementary Fig. 3. (**b**) Magnified view of the overlay of **MRL-871** (orange) and **MRL-367** (cyan) in the novel allosteric binding pocket of RORγt (overall root mean squared deviation (r.m.s.d) 0.219). (**c**) Magnified view of overlay of **MRL-367** (cyan) and **MRL-673** (yellow) in the novel allosteric binding pocket of RORγt (overall r.m.s.d 0.38). **MRL-673** is a racemic mixture and the two isomers are depicted. Arrows highlight repositioning of the side chains of residues Tyr502 and Phe498 and the accompanying shift in the backbone (for example, Ala497) on binding **MRL-673**.

We explored the synthetic optimization of **MRL-871** to establish structure–activity relationships around the indazole series. These compounds were tested in biochemical assays, including the competition assay using the fluorescent-labelled allosteric probe, cellular chimeric receptor reporter assays in HEK-293 cells and Th17 differentiation/IL17a production assays in primary human peripheral blood mononuclear cells (PBMCs), as well as structural studies (Fig. 3). Table 2 describes several informative examples. **MRL-299** features two additional fluoro groups, one at the indazole ring and one at the phenyl substituent. **MRL-367** contains additional polar functionality—the phenyl substituent contains a hydroxyl substituent ortho to the carboxylic acid, and the C-4 of the indazole is replaced by nitrogen. In the case of **MRL-673**, the benzoic acid functionality is saturated, and the carbon atom adjacent to the carboxylate is alkylated, resulting in a tetrasubstituted carbon centre. This modification induces a displacement of the carboxyl group and highlights the importance of the polar interactions in the binding site.

The additional fluorine groups (**MRL-299**) and the polar functionalities (**MRL-367**) were both important for RORγt-binding potency and functional inhibition (Table 2). In particular, **MRL-367** exhibited enhanced potency in the cofactor displacement and direct binding assay, as well as increased inhibitory activity in a luciferase reporter assay and in functional inhibition of IL-17 production. To insure that the functional activity of the indazole series is attributable to inhibition of RORγt, **MRL-299** was tested against a commercially available panel of cell-based NR reporter assays (Supplementary Table 1). Across this panel of NR assays, **MRL-299** was >100-fold selective for RORγt. The only significant off-target activity was against PPARγ (PPARγ activity was also recently reported for a structurally similar series of molecules^29^), which was de-risked (Supplementary Note 1).

Structural elucidation of these compounds reveals that all antagonists occupy the same allosteric-binding pocket as **MRL-871** (Fig. 3). Detailed comparison of, for example, **MRL-871** and **MRL-367** (Fig. 3b) reveals that both polar molecular additions to the scaffold are fully tolerated and lead only to very minor changes in the surrounding amino-acid orientations. **MRL-673**, in contrast, exhibits 10-fold weaker potency in both the biochemical and cellular assays relative to **MRL-367**. Structural comparison of, for example, **MRL-367** and **MRL-673** (Fig. 3c) reveals that, while these compounds bind the same allosteric site, the position of the carboxylate ring of **MRL-673** distorts the binding pocket. The modification induces a displacement of the carboxyl group and highlights the importance of the polar interactions in the binding site. Although the carboxylate of **MRL-673** makes the same number of polar contacts with RORγt as **MRL-367**, the methylation induces the reorientation of the side chains of Phe498 and Tyr502. This shift in the side chains and the accompanying shifts in the backbone of the C-terminal residues of Ala497 and Phe498, explains the lower receptor affinity and functional inhibitory potency.

## Discussion

NR drug development has been based primarily on the ability of NRs to bind ligands at the highly conserved hydrophobic orthosteric pocket of the LBD, which is also the binding site of endogenous ligands^1^,^18^. Compared with unbound NRs, the binding of an agonist induces a conformational change that stabilizes the positioning of H12 and AF-2 domain, resulting in coactivator recruitment. The binding of antagonist or inverse agonist ligands typically destabilizes H12 and the AF-2 domain (possibly unfolding it) resulting in a lack of coactivator or enhanced corepressor recruitment, ultimately resulting in transcriptional inhibition at specific loci. Numerous successful drugs targeting NRs have been developed, all working via this mechanism, but increasing resistance against certain cancer therapy oriented NR antagonists, as well as the challenge of targeting orphan receptors has increased the need for alternative site modulators of NRs^19^,^20^,^21^. Similar to, for example, the efforts to target allosteric sites in protein kinases^30^, allosteric NR modulators may differentiate favourably versus orthosteric ligands. Potential advantages of allosteric NR inhibitors are enhanced selectivity, no competition with increasing endogenous ligands during pathological conditions, and no sensitivity to agonist/antagonist switching due to mutations.

**Figure 4:**
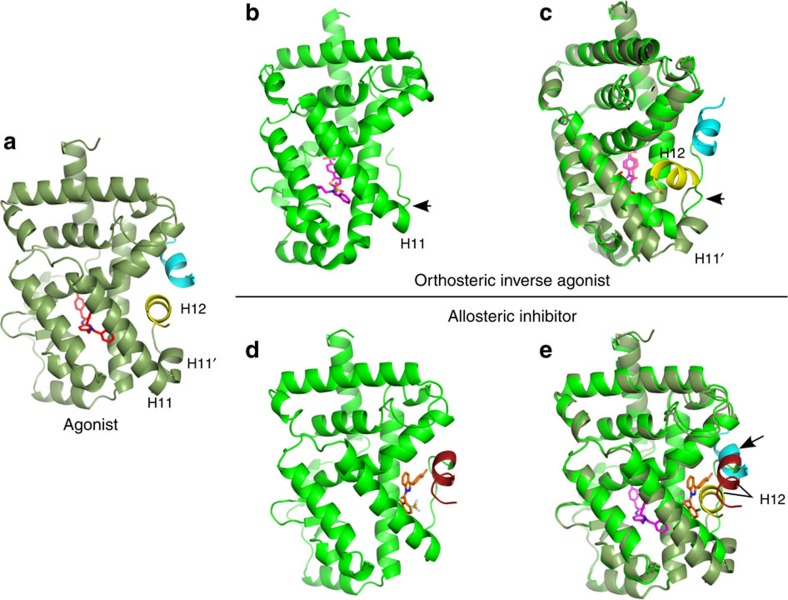
Molecular mechanism of allosteric RORγt modulation. (**a**) Agonistic conformation of RORγt^31^ (PDB entry 4NIE) showing the position of the agonistic ligand (red) in the classical orthosteric ligand binding site. Helices 11′ and 12 (yellow) of the LBD are folded in a stable agonistic conformation, supporting binding of the LXXLL cofactor peptide (blue). (**b**) Antagonistic conformation of RORγt^14^ (PDB entry 4QM0) induced by a classical inverse agonist (purple) in the classical orthosteric ligand-binding site. Helices 11 and 12 of the LBD are unstructured (arrow). (**c**) Superposition of **a** and **b**. When H12 is disordered, the LXXLL cofactor peptides lack a complementary surface for binding to RORγt (**d**) Antagonistic conformation of RORγt, induced by allosteric modulator **MRL-871** (orange). **MRL-871** makes direct contact with H12 (brown) at the allosteric site in the RORγt LBD and stabilizes the folding of H12. (**e**) Superposition of **a** and **d**. The binding of the allosteric modulator **MRL-871** results in unfolding of helix 11′ and shifts the position of H12 to directly block cofactor peptide binding. For a structural comparison with agonist hydroxycholesterol liganded RORγt see Supplementary Fig. 5.

The recently reported crystal structure of RORγt with a potent tertiary amine orthosteric agonist revealed the coactivator-binding site on the LBD, in a classical NR H12 switch agonist mode and bound to a cofactor peptide motif (Fig. 4a)^31^. The new crystal structures of RORγt reported here with **MRL-871** and its analogues (Fig. 3) reveal that these indazole modulators bind to a receptor position normally occupied by H12 in the non-liganded or agonist ligand-bound conformation. The final orientation of H12 in the presence of the novel indazole modulators is such that the classical binding surface for the cofactor LXXLL motif is not only modified, but actively blocked (Fig. 4e). This overall orientation effectively antagonizes cofactor binding to RORγt. The consequence of this allosteric modulator-mediated refolding of RORγt is functional inhibition as evidenced by biochemical and cellular studies. The unique mode of allosteric binding reported thus provides a structural rationale for targeting NRs with small molecules in an orthogonal manner that does not require competition with canonical ligands.

**Figure 5:**
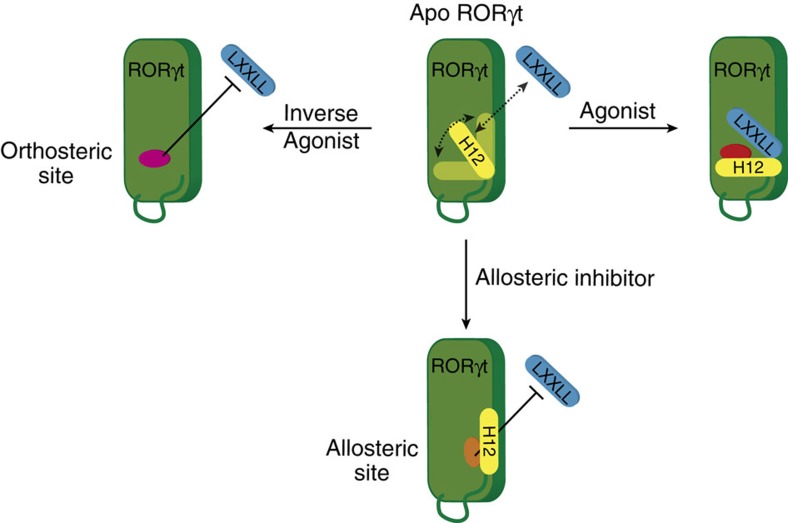
Schematic model of the novel molecular mechanism of allosteric RORγt inhibition regarding H12 folding and positioning. In the apo form, H12 is dynamic and thus limits LXXLL cofactor binding. An inverse agonist destabilizes H12 and lowers LXXLL binding. Agonist binding stabilizes H12 in a optimal conformation for LXXLL binding. Binding at the novel allosteric site, reorients the H12 of RORγt and actively blocks LXXLL binding.

The X-ray structures demonstrate that these indazoles all occupy the novel allosteric binding pocket. Reported crystal structures with orthosteric inverse agonists, binding to the canonical site of RORγt, lack clear structural data for the position of the H12 and the resulting cofactor-binding site (for example, Fig. 1c with T0901317 and Fig. 4b). Synthetic RORγt inverse agonists T0901317 (ref. 27) and tertiary sulfonamides^26^ bind at the canonical orthosteric NR ligand-binding pocket. Their binding distorts the structure of the LBD, leading to helices 11′ and 12 being unstructured in the crystallized protein (Fig. 4b). A superposition of the RORγt structures with an inverse agonist in the orthosteric site and an indazole modulator in the allosteric site reveals the additional benefit of the novel site (Fig. 1c; Supplementary Fig. 4). Whereas both compounds induce the partial unfolding of helix 11′ (ref. 32), **MRL-871** subsequently also stabilizes the folding of H12 via direct interactions. The unfolded helix 11′ spans the distance to the displaced H12 N terminus (Fig. 4d). This results in RORγt taking on a stably folded antagonistic state and directly blocking cofactor peptide binding (Fig. 4e). These antagonists thus induce a conformational change in the RORγt LBD, which blocks cofactor binding through the stabilization of the H12 subdomain in an unprecedented folded state (Fig. 5).

In summary, we demonstrate the structural and functional elucidation of an unprecedented NR allosteric inhibitory mechanism, based on highly potent small-molecule modulators for RORγt. This is the first allosteric binding pocket with highly potent small drug-like molecules, functionally active in Th17 differentiation/IL17a production inhibition. This allosteric inhibitory mechanism offers great advantages for NR drug development, including selectivity, independence of endogenous ligands and less responsive to point mutations within the orthosteric ligand binding site. RORγt regulates a variety of physiological processes and has emerged as a highly promising drug target for autoimmune diseases. The discovery of this novel allosteric NR pocket and concomitant selective molecules that target this site offers a new strategic opportunity to develop agonist-independent therapeutics for Th17-mediated autoimmune disorders.

## Methods

### Synthesis of indazole series

For the synthesis of the 3-iodo-1*H*-indazol-1-yl(phenyl)methanone, thionyl chloride was added dropwise to the appropriate benzoic acid in an oven dried flask. The reaction mixture was stirred at 75 °C overnight. After removal of the thionyl chloride under reduced pressure, the benzoyl chloride was dissolved in dry CH_2_Cl_2_. To this solution, 3-iodo-1*H*-indazole and DMAP were added. Finally, triethylamine was added dropwise and the mixture was stirred at room temperature for 24 h. The reaction mixture was then diluted with H_2_O and CH_2_Cl_2_. The layers were separated and the aqueous layer was washed with CH_2_Cl_2_. The combined organic layers were washed with brine, dried over Na_2_SO_4_, filtered and evaporated. The crude material was purified via column chromatography.

For the cross-coupling reaction the appropriate 3-iodo-1*H*-indazole, (4-(methoxycarbonyl)-phenyl)boronic acid, Pd(PPh_3_)_4_ and CH_3_CO_2_K were dissolved in dioxane/H_2_O (5:1 v/v) in a Schlenk tube and the reaction was stirred at 90 °C. After 3 h, the mixture was allowed to cool to room temperature and diluted with CH_2_Cl_2_ and H_2_O. The organic layer was washed with brine, dried over Na_2_SO_4_, filtered and concentrated. The obtained crude material was purified via column chromatography.

To obtain the free acid, LiOH · H_2_O was added to a solution of the methyl ester in THF/H_2_O. The reaction was stirred at room temperature. After 24 h, the reaction was diluted with H_2_O, neutralized with acetic acid (∼pH 4) and extracted with CH_2_Cl_2_. The combined organic layers were washed with brine, dried over Na_2_SO_4_, filtered and evaporated. The final products (for example, **MRL-871**) were purified via preparative liquid chromatography-mass spectrometry (LCMS). For complete synthesis and characterization data see Supplementary Methods.

### HTRF assay

The human RORγt LBD used for the HTRF assay was expressed as a His_6_-tag fusion protein from the pET15b expression vector in *Escherichia coli* BL21(DE3) cells. Cells transformed with this vector were grown in 2 × YT medium supplemented with ampicillin until an OD_600_=0.7 was reached. Protein expression was then induced with 0.1 mM isopropyl-b-d-thiogalactoside (IPTG). After incubation for 16 h at 16 °C, cell cultures were collected by centrifugation. The cells were lysed via sonication and the protein was purified via Ni^2+^-affinity column chromatography.

The homogeneous TR-FRET assays were performed in triplicate with 20 nM His_6_-RORγt and 100 nM biotin labelled cofactor peptide. Terbium-labelled anti-His antibody (cat. no: 610HATAA, 0.71 nM) and D2-labelled streptavidin (cat. no: 610SADLA, 41.7 nM) were used at recommended concentrations by the supplier Cisbio Bioassays. Assay buffer contained 100 mM HEPES (pH 7.5), 100 mM NaCl, 5 mM dithiothreitol (DTT) and 0.1% bovine serum albumin. The plates (white 384-well plates Greiner Bio-One) were incubated for 2 h at 4 °C before the FRET ratio was measured in a Tecan Infinite F500 plate reader. The data were analysed using Origin software. Dose–response curves were fitted following:


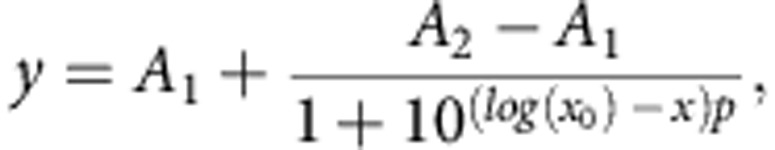


where *A*_1_ is the botom asymptote, *A*_2_ is the top asymptote and *p* is the Hill slope.

### RORγt-LBD SRC-1 cofactor FRET assay

RORγt LBDs of from human (Genbank accession number NP_005051 aa259–518) and mouse (Genbank accession number NP_035411 aa236–516) were amplified by PCR and cloned into pET47b to include the sequence MAHHHHHHHHENLYFQGTPE N terminal to the first residue of the RORγt-coding sequence. Proteins were expressed in BL21(DE3) *E. coli* grown in LB media. Expression was induced with 0.1 mM IPTG and allowed to continue for 16 h at 16 °C. Cells were lysed in 50 mM Tris, 300 mM NaCl, 10 mM imidazole, 5% glycerol, 10 mM beta-mercaptoethanol, 1% Triton X-100 and 0.2 mM PMSF, pH 8.5 using a microfluidizer. After removal of cellular debris by centrfugation, lysates were loaded onto Ni-NDA resin and washed with 50 mM Tris, 300 mM NaCl, 20 mM imidazole, 5% glycerol and 10 mM beta-mercaptoethanol at pH 8.5. The column was washed with the same buffer containing 60 mM imidazole, and protein was eluted with the above buffer containing 250 mM imidazole. The proteins were dialysed into 50 mM Tris, 100 mM NaCl, 5% glycerol and 1 mM DTT, pH 8.5 for storage at −80 °C. The potency of small-molecule RORγt ligands was then assessed by monitoring their effect on the association of a LXXLL-motif-containing SRC-1 peptide. Compound (10 mM dimethylsulphoxide (DMSO) stock) was serially diluted in threefold steps using an Agilent Bravo liquid handler. Diluted compound or DMSO (25 nl) was transferred into a black Greiner 384-well plate (Cat#781076) using a LabCyte Echo acoustic dispenser. To each well of the plate was added 15 μl of 3.75 nM RORγt-LBD in receptor buffer (50 mM Tris-HCl pH 7.0, 50 mM potassium chloride, 1 mM EDTA, 0.1% delipidated bovine serum albumin, 1 mM DTT, 1.25 nM Anti-His W1024 Europium chelate antibody (PerkinElmer) and 3% (v/v) of lysate from ∼24,000 Sf9 cells). Compounds were allowed to incubate with receptor for 15 min and then 5 μl of peptide in detection buffer or detection buffer alone were added. Detection buffer (5 × ) consists of 50 mM Tris-HCL pH 7.0, 50 mM potassium chloride, 1 mM EDTA, 0.1% delipidated bovine serum albumin, 1 mM DTT and 20 nM streptavidin-APC (PerkinElmer). When peptide (Biotin-SPSSHSSLTERHKILHRLLQEGSP) was included, its concentration in the 5 × stock was 250 nM. The plate was then incubated overnight at 4 °C. The following morning the plate was warmed to room temperature and read using an Envision plate reader (PerkinElmer). TR-FRET signal was defined as the ratio of the fluorescence emission at 615 to 665 nm following excitation at 337 nm. The per cent activity of each dilution was determined as the ratio of background corrected signal to the background corrected signal of wells receiving only DMSO. IC_50_ values were determined by fitting per cent activity data to a four-parameter logistic dose–response equation in GraphPad Prism (GraphPad Software).

### RORγt-LBD AlexaFluor-647-labelled MRL-871 competitive binding assay

As an alternate assessment of inhibitor potency MRL-871 was covalently labelled with Alexa Fluor-647. To determine the intrinsic affinity of the probe, the compound (5 mM DMSO stock) was serially diluted in threefold steps using an Agilent Bravo liquid handler. Diluted probe or DMSO (25 nl) were transferred into a black Greiner 384-well plate (Cat#781076) using a LabCyte Echo acoustic dispenser. To each well of the plate was added 15 μl of RORγ-LBD at 1.6, 3.33 and 6.65 nM in receptor buffer or receptor buffer alone. The plate was then incubated for 15 min at room temperature. Subsequently, 5 μl of receptor buffer with 1.2 nM Anti-His W1024 Europium chelate antibody (PerkinElmer) was added and the TR-FRET signal measured as in the SRC-1 cofactor recruitment assay described above. TR-FRET intensity in the presence of RORγt was corrected for background and the *K*_D_ determined using the One-site specific binding equation in Graph Pad Prism. To assess the potency of unlabelled compounds, a similar assay format was utilized except that 100 nM probe was preincubated with the RORγt-LBD for 15 min before addition to serially diluted compounds. IC_50_ values were determined by fitting per cent activity data to a four-parameter logistic dose–response equation in GraphPad Prism.

### RORγt crystallization and structure determination in complex with MRL-871

A pCDFDuet-1 expression vector encoding the RORγt LBD (residues 265–507) with an N-terminal StrepII-SUMO-tag was transformed by heat shock into Nico21(DE3) *E. coli* cells (NEB). Single colonies were used to inoculate three pre-cultures of 30 ml LB containing 100 μg ml^−1^ streptomycin. After overnight incubation at 37 °C, each pre-culture was transferred to 1.5 l of ZYP5052 medium^33^ without lactose (making ZYP505), but with 100 μg ml^−1^ streptomycin and 0.1 % antifoam SE-15 (Sigma-Aldrich). These cultures were incubated further until they reached an OD_600nm_=4. At that point, the temperature was decreased to 18 °C, protein expression was induced by adding IPTG to a final concentration of 200 μM and the cultures were grown for 24 h more. The cells were collected by centrifugation and were dissolved in lysis buffer (20 mM Tris pH 7.5, 300 mM NaCl, 5 mM MgCl_2_, 2 mM 2-mercapto-ethanol and 10 μg ml^−1^ DNase I (Sigma-Aldrich)) at 10 g per 100 ml. After cell lysis using an Emulsiflex-C3 homogeniser (Avestin), the cell lysate was cleared by centrifugation (at 4 °C) and the supernatant was loaded on a column consisting of 10 ml Strep-Tactin Superflow High Capacity resin (IBA). The fusion protein was eluted from the resin with three column volumes elution buffer (20 mM Tris, 300 mM NaCl, 2 mM 2-mercapto-ethanol and 2.5 mM desthiobiotin at pH 7.5) and the StrepII-SUMO-tag was removed by adding dtUD1 SUMO protease^34^. Next, the protein mixture was concentrated using an Amicon ultra-centrifugation device with a 3-kDa cutoff (Millipore) and loaded on a Superdex 75 pg 16/60 size-exclusion column (GE Life Sciences). Here the cleaved StrepII-SUMO-tag and RORγt LBD co-eluted. To separate them, the size-exclusion chromatography (SEC) fractions were combined and loaded for a second time over the regenerated Strep-Tactin column. Finally, pure RORγt was collected in the flow-through and concentrated in presence of **MRL-871** to 8 mg ml^−1^.

Before crystallization, RORγt was mixed with an additional 1 equivalent of ligand. The crystals were grown at room temperature using the sitting drop vapour diffusion method. Optimal crystals were grown in a week by mixing 1 μl protein solution with the same volume of a crystallization condition containing 0.2 M MgCl_2_, 0.1 M Tris, pH 8.5 and 7% w/v polyethylene glycol 6 K. The well reservoir was filled with 1 ml of the crystallization condition. Crystals were cryoprotected in the mother liquor supplemented with 20 % glycerol and flash cooled in liquid nitrogen. Diffraction data were collected at 100 K at the PX II beamline at the SLS synchrotron (Villigen, Switzerland). iMOSFLM^35^ and AIMLESS^36^ of the CCP4 suite^37^ were used for integration and scaling, respectively. The structure was phased by molecular replacement using PDB ID 4NIE^31^ as search model in Phaser^38^. Ligand restraints were generated using Grade (Global Phasing, version 1.2.8) and CCDC Mogul^39^. Coot^40^ and phenix.refine^41^,^42^ were used in alternating cycles of model building and refinement. The quality of the final model was evaluated using MolProbity^43^. Figures were created using PyMOL (The PyMOL Molecular Graphics System, Version 1.7. Schrödinger, LLC) and LigPlot+ (ref. 44). Structure coordinates were deposited in the Protein Data Bank (PDB code 4YPQ). For a stereo image of a portion of the electron density map of the co-crystal structure of RORγt with **MRL-871** see Supplementary Fig. 6.

### RORγt crystallization and structure determination in complex with MRL-871, MRL-299, MRL-367 and MRL-673

A pETDuet-1 expression vector encoding the RORγt LBD (residues 267–507) with an N-terminal His Tag was transformed by heat shock into *E. c*oli BL21-Gold(DE3)pLysS (Catalog #230134, Agilent Technologies, USA). LB Amp containing starter cultures were inoculated with single colonies of the day before freshly transformed cells. Starter cultures were shaken overnight. The next day 1.0 l LB Amp expression cultures were inoculated to an OD_600nm_=0.05 AU and incubated in 2.5 l Ultra Yield Flasks (HTS Labs, USA) at 30 °C. At an OD_600nm_ of about 0.8 AU cultures were cooled down quickly to 15 °C. After 20 min on reaching 15 °C, RORγt expression was induced by the addition of 0.25 mM IPTG. Cells were collected 16 h after induction, resuspended and sonicated in pre-cooled buffer A (50 mM Na_2_KPO_4_, 500 mM NaCl, 10% (v/v) glycerol, 3 mM TCEP, 0.1% Tween20 and 2 mM CHAPS, pH 8.0) with a ratio of cells:buffer of 1:5 (w/v). Before cell disruption, two tablets of ‘Protease Inhibitor Cocktail, EDTA-free' (Roche), 20 mg of lysozyme and 1800 U of benzonase were added per 100 ml of lysis buffer and incubated on ice for 30 min under gentle stirring. The cell paste was fluidized with two passes through a fluidizer at 12,000 p.s.i. The cell lysate was clarified by centrifugation at 48,000*g* for 40 min, the supernatant was collected and the pH of the lysate was adjusted to 7.5. The sample was loaded on to a pre-chilled HisTrap FF column (buffer A). To prepare the magnetic beads, Ni SepFast MAG beads were washed four times with 10 bead volumes of water and equilibrated in 20 bead volumes of buffer (50 mM phosphate, 500 mM NaCl, 2 mM CHAPS, 10 % glycerol, 3 mM TCEP, 0.1 % Tween20 and 1 tablet of complete protease inhibitor per 50 ml, pH 8.0). The supernatant obtained was removed, the beads were washed with 20 bead volumes of buffer (50 mM phosphate, 200 mM NaCl, 20 mM imidazole, 10% glycerol, 2 mM CHAPS, 3 mM TCEP, 0.1 % Tween20 and 1 tablet of complete protease inhibotor per 50 ml, pH 8.0). RORγt protein was eluted from the column with (15–100%) gradient of buffer (50 mM phosphate pH 8, 200 mM NaCl, 2 mM CHAPS, 10% glycerol, 300 mM imidazole, 3 mM TCEP and 0.1% Tween20). The His-tag was cleaved by adding tobacco etch virus (TEV) protease and by incubating overnight at 4 °C. Efficiency of the cleavage reaction was examined by SDS–polyacrylamide gel electrophoresis analysis. The samples were re-run over a new HisTtrap column and the flow-through, containing RORγt samples, were collected and concentrated to 2 mg ml^−1^ and flash frozen for storage.

Protein–ligand complex was prepared by mixing 1:10 molar ratio (protein:ligand) and incubated for 2 h on ice. The sample was hard spun at 20,000*g* for 20 min and was loaded onto pre-equilibrated (SEC buffer 20 mM Tris, pH 8.5, 100 mM NaCl and 2 mM DTT) S-200 SEC column. The RORγt–ligand complex was concentrated to 10–15 mg ml^−1^ before crystallization. Crystals of RORγt in complex with ligands were grown using hanging drop vapour diffusion set-ups. A mesure of 0.2 μl of protein solution (13 mg ml^−1^ in 20 mM Tris, 100 mM NaCl and 5 mM DTT, pH 8.5) was mixed with 0.2 μl of reservoir solution (1.2–1.8 M ammonium sulfate and 0.1 M Tris/HCl, pH 8.0–9.0) and equilibrated over 0.5 ml of reservoir solution at 20 °C. Well-diffracting crystals appeared within 2 days. The mounted crystal was flash frozen with 20% glycerol as cryo protectant in the mother liquor. Data were collected from frozen crystals at 100 K in the facilities of the Industrial Macromolecular Crystallography Association, at the Advanced Photon Source located at Argonne National Laboratories in Argonne, IL. Data were reduced and scaled using HKL2000 (HKL Research, Charlottesville, VA). The structure was determined by molecular replacement using molrep (CCP4) and the RORγt hydroxycholesterol structure (PDB ID: 3L0J)^24^ as the search model. The structure was refined using Buster (Global Phasing); model building was performed with coot (CCP4) and further refined with Buster. Figures were made with PyMol (Schrödinger). Structure coordinates were deposited in the Protein Data Bank (PDB codes 5C4O, 5C4U, 5C4S and5C4T).

### Chimeric RORγt-GAL4 reporter assay

The coding sequence of RORγt aa97–518 was cloned in frame with the DNA-binding domain of the yeast GAL4 protein within the CMV-promoter-driven pCDNA3.1 vector. This vector, along with the GAL4 UAS-luciferase reporter vector pGL4.31 (Promega), was used to transfect HEK293T cells. Briefly, 1 × 10^7^ cells in 10 ml of DMEM high-glucose media with 10% fetal bovine serum (FBS) were transfected with a mixture consisting of 10 μg of each plasmid and 60 μl of TransIT-293 (Mirus Bio) in 1.5 ml of Optimem (Invitrogen). Following transfection, cells were transferred to one T75 flask and incubated overnight at 37 °C and 5% CO_2_. Compound dilutions are prepared as above and 50 nl was transferred to a 384-well Greiner white tissue-culture-treated plate (catalog #781080) using an Echo acoustic dispenser (LabCyte). Cells were collected and resuspended at 0.8 × 10^6^ cells per ml in DMEM high-glucose media with 10% FBS. To each well of the plate was added 25 μl of cell suspension and the cells incubated overnight at 37 °C and 5% CO_2_. After 20–22 h, the plates were brought to room temperature and 25 μl of Steady-Glo luciferase reagent (Promega) was added to each well. The luminescent signal was measured on an Envision plate reader.

### Quantitative IL17a RT–PCR

EL4 cells (Sigma-Aldrich) were grown in DMEM (Gibco). At 24 h after the cells were seeded onto a 12-well plate, the cells were treated with ligands **1–3** (from 10 mM stock in DMSO) or DMSO. After 24 h, the cells were collected and RNA was isolated using a RNeasy Plus Micro Kit (Qiagen) and reverse transcribed using the iScrip cDNA biosynthesis kit (Bio-Rad). Quantitative RT–PCR was performed to analyse mRNA levels of mouse IL17a levels using SYBR green technology (Bio-Rad) on a CFX Real-Time System (Bio-Rad). Primer sequences used for *IL17a*:^45^ Fw: 5′-ctccagaaggccctcagactac-3′, Rev: 5′-ctgtgtcaatgcggagggaaagct-3′ and *Gapdh*: Fw: 5′-ggtggacctcatggcctaca-3′, Rev: 5′-ctctcttgctcagtgtccttgct-3′. The level of IL17a mRNA expression was normalized to that of Gapdh expression. All data are expressed as the mean±s.e.m. (*n*=6). Statistical analysis was performed using an one-way analysis of variance comparing against the DMSO control following Dunnett *post hoc* test.

### PBMC Th17 polarization and IL-17 production assay

Test compounds were prepared as 10 mM stocks in DMSO and serially diluted 1:3 to provide an eight-concentration titration. The compounds (200 nl of each dilution) were acoustically dispensed into a 96-well Costar 3912 assay plate. Frozen human PBMCs from an anonymous healthy donor were obtained commercially and were diluted to a density of 5 × 10^5^ cells per ml with growth media (RPMI 1640/ 10% FBS/pen/strep). Stimulatory cytokines were added to final concentrations of 25 ng ml^−1^ IL-1B, 10 ng ml^−1^ IL-23, 0.5 ng ml^−1^ IL-2 and 10 ng ml^−1^ IL-6 (all cytokines from R&D Systems). In addition, T-Activator CD3/28 Dynabeads (Invitrogen) were added to a concentration of 100,000 beads per ml. The stimulated cells were immediately dispensed into the assay plate containing serially diluted compound at a volume of 200 μl cells per well. Cell plates were then incubated at 37 °C and 5% CO_2_ for 4 days. Culture media (100 μl) was collected from each well and IL-17 expression was measured by enzyme-linked immunosorbent assay (R&D Systems) according to the manufacturer's instructions. Cell viability was assessed by the addition of 100 μl of CellTiter-Glo (Promega) to each well of the cell assay plate followed by luminescence detection on an Envision plate reader (PerkinElmer).

## Additional information

**Accession codes:** Coordinates and structure factors for the RORγt bound to **MRL-871**, **MRL-299 MRL-367** and **MRL-673** have been deposited in the Protein Data Bank under accession codes 4YPQ, 5C4O, 5C4U, 5C4S and 5C4T.

**How to cite this article:** Scheepstra, M. *et al.* Identification of an allosteric binding site for RORγt inhibition. *Nat. Commun.* 6:8833 doi: 10.1038/ncomms9833 (2015).

## Supplementary Material

Supplementary Information Supplementary Figures 1-6, Supplementary Table 1, Supplementary Note 1, Supplementary Methods and Supplementary Reference

## Figures and Tables

**Table 1:** Data collection and refinement statistics (molecular replacement).

**PDB ID**	**4YPQ**	**5C4O**	**5C4U**	**5C4S**	**5C4T**
Ligand name	**MRL-871**	**MRL-871**	**MRL-367**	**MRL-299**	**MRL-673**
					
*Data collection*					
Space group	R32:H	P 61 2 2	P 61 2 2	P 61 2 2	P 61 2 2
Cell dimensions					
*a*, *b*, *c* (Å)	173.8, 173.8, 67.2	108.5, 108.5, 104.7	108.1, 108.1, 106.5	108.4, 108.4, 106.3	107.3, 107.3, 100.4
*α*, *β*, *γ* (°)	90, 90, 120	90, 90, 120	90, 90, 120	90, 90, 120	90, 90, 120
Resolution (Å)	35.47–2.32 (2.40–2.32)*	69.91–2.24 (2.32–2.24)	93.6–2.08 (2.154–2.08)	93.92–2.23 (2.31–2.23)	92.9–1.77 (1.836–1.77)
*R*_sym_	0.121 (0.886)	0.047 (1.45)	0.0420 (1.236)	0.056 (1.402)	0.0565 (1.239)
*I*/σ*I*	12.1 (2.7)	35.91 (2.57)	38.87 (2.62)	30.03 (2.51)	29.46 (2.61)
Completeness (%)	100.0 (100.0)	99.94 (99.89)	99.78 (99.36)	99.99 (100.0)	99.99 (100.00)
Redundancy	11.7 (11.6)	16.0 (15.8)	19.3 (19.9)	18.8 (19.2)	19.1 (19.7)
					
*Refinement*					
Resolution (Å)	35.47–2.32	69.91–2.24	93.6–2.08	93.9–2.23	92.9–1.77
No. reflections	16882	17932	22579	18547	33582
*R*_work_/*R*_free_	0.174/0.227	0.227/0.266	0.228/0.273	0.218/0.261	0.192/0.223
No. atoms					
Protein	1987	1996	1994	1987	1985
Ligand/ion	32	60	51	45	118
Water	130	14	23	18	101
*B*-factors					
Protein	45.70	67.90	59.80	67.9	39.6
Ligand/ion	41.50	102.5	57.70	64.1	50.50
Water	47.30	64.20	57.80	63.9	45.30
R.m.s. deviations					
Bond lengths (Å)	0.008	0.014	0.014	0.014	0.015
Bond angles (°)	1.04	1.78	1.81	1.75	1.64

R.m.s., root mean squared.

^*^Values in parentheses are for highest-resolution shell.

**Table 2:**
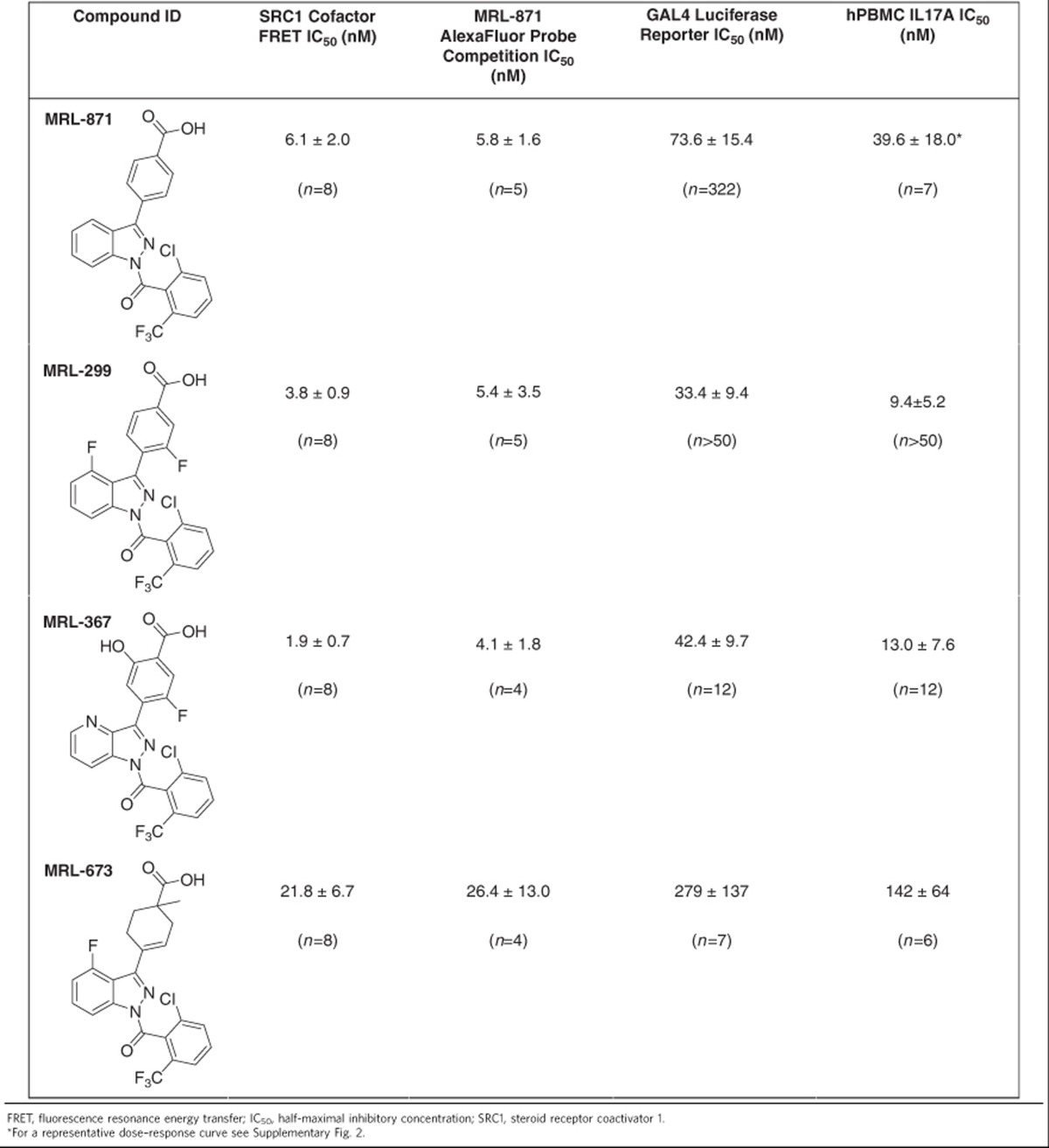
Potency (mean±s.d.) of selected RORγt allosteric inverse agonists in biochemical and cellular assays.

## References

[b1] Gronemeyer H., Gustafsson J.-Å. & Laudet V. Principles for modulation of the nuclear receptor superfamily. Nat. Rev. Drug Discov. 3, 950–964 (2004). 15520817 10.1038/nrd1551

[b2] Overington J. P., Al-Lazikani B. & Hopkins A. L. How many drug targets are there? Nat. Rev. Drug Discov. 5, 993–996 (2006). 17139284 10.1038/nrd2199

[b3] Solt L. A. & Burris T. P. Action of RORs and their ligands in (patho)physiology. Trends Endocrinol. Metab. 23, 619–627 (2012). 22789990 10.1016/j.tem.2012.05.012 PMC3500583

[b4] Solt L. A. *et al.* Suppression of TH17 differentiation and autoimmunity by a synthetic ROR ligand. Nature 472, 491–494 (2011). 21499262 10.1038/nature10075 PMC3148894

[b5] Xu T. *et al.* Ursolic acid suppresses interleukin-17 (IL-17) production by selectively antagonizing the function of RORgamma t protein. J. Biol. Chem. 286, 22707–22710 (2011). 21566134 10.1074/jbc.C111.250407 PMC3123037

[b6] Fujita-Sato S. *et al.* Structural basis of digoxin that antagonizes RORgamma t receptor activity and suppresses Th17 cell differentiation and interleukin (IL)-17 production. J. Biol. Chem. 286, 31409–31417 (2011). 21733845 10.1074/jbc.M111.254003 PMC3173075

[b7] Yang Y. *et al.* Impact of suppressing retinoic acid-related orphan receptor gamma t (ROR)γt in ameliorating central nervous system autoimmunity. Clin. Exp. Immunol. 179, 108–118 (2015). 25142403 10.1111/cei.12441 PMC4260903

[b8] Kanai T., Mikami Y., Sujino T., Hisamatsu T. & Hibi T. RORγt-dependent IL-17A-producing cells in the pathogenesis of intestinal inflammation. Mucosal Immunol. 5, 240–247 (2012). 22354322 10.1038/mi.2012.6

[b9] Huang Z., Xie H., Wang R. & Sun Z. Retinoid-related orphan receptor γt is a potential therapeutic target for controlling inflammatory autoimmunity. Expert Opin. Ther. Targets 11, 737–743 (2007). 17504012 10.1517/14728222.11.6.737

[b10] Isono F., Fujita-Sato S. & Ito S. Inhibiting RORγt/Th17 axis for autoimmune disorders. Drug Discov Today 19, 1205–1211 (2014). 24792721 10.1016/j.drudis.2014.04.012

[b11] He Y. W. *et al.* Down-regulation of the orphan nuclear receptor ROR gamma t is essential for T lymphocyte maturation. J. Immunol. 164, 5668–5674 (2000). 10820242 10.4049/jimmunol.164.11.5668

[b12] Yang X. O. *et al.* T Helper 17 lineage differentiation is programmed by orphan nuclear receptors RORα and RORγ. Immunity 28, 29–39 (2008). 18164222 10.1016/j.immuni.2007.11.016 PMC2587175

[b13] Fauber B. P. & Magnuson S. Modulators of the nuclear receptor retinoic acid receptor-related orphan receptor-γ (RORγ or RORc). J. Med. Chem. 57, 5871–5892 (2014). 24502334 10.1021/jm401901d

[b14] Fauber B. P. *et al.* Reduction in lipophilicity improved the solubility, plasma–protein binding, and permeability of tertiary sulfonamide RORc inverse agonists. Bioorg. Med. Chem. Lett. 24, 3891–3897 (2014). 25017032 10.1016/j.bmcl.2014.06.048

[b15] Brzozowski A. M. *et al.* Molecular basis of agonism and antagonism in the oestrogen receptor. Nature 389, 753–758 (1997). 9338790 10.1038/39645

[b16] Nichols M., Rientjes J. M. & Stewart A. F. Different positioning of the ligand-binding domain helix 12 and the F domain of the estrogen receptor accounts for functional differences between agonists and antagonists. EMBO J. 17, 765–773 (1998). 9451001 10.1093/emboj/17.3.765 PMC1170425

[b17] Heery D. M., Kalkhoven E., Hoare S. & Parker M. G. A signature motif in transcriptional co-activators mediates binding to nuclear receptors. Nature 387, 733–736 (1997). 9192902 10.1038/42750

[b18] Nagy L. & Schwabe J. W. Mechanism of the nuclear receptor molecular switch. Trends Biochem. Sci. 29, 317–324 (2004). 15276186 10.1016/j.tibs.2004.04.006

[b19] Moore T. W., Mayne C. G. & Katzenellenbogen J. A. Minireview: Not picking pockets: nuclear receptor alternate-site modulators (NRAMs). Mol. Endocrinol. 24, 683–695 (2010). 19933380 10.1210/me.2009-0362 PMC2852352

[b20] Hughes T. S. *et al.* An alternate binding site for PPARγ ligands. Nat. Commun. 5, 3571 (2014). 24705063 10.1038/ncomms4571 PMC4070320

[b21] Scheepstra M. *et al.* A natural-product switch for a dynamic protein interface. Angew. Chem. Int. Ed. Engl. 53, 6443–6448 (2014). 24821627 10.1002/anie.201403773

[b22] Karstens W. F. J. *et al.* RORgammaT Inhibitors. PCT Int. Appl. WO 2012/106995 (2012).

[b23] Harris J. M., Lau P., Chen S. L. & Muscat G. E. O. Characterization of the retinoid orphan-related receptor-alpha coactivator binding interface: a structural basis for ligand-independent transcription. Mol. Endocrinol. 16, 998–1012 (2002). 11981035 10.1210/mend.16.5.0837

[b24] Jin L. *et al.* Structural basis for hydroxycholesterols as natural ligands of orphan nuclear receptor ROR? Mol. Endocrinol. 24, 923–929 (2010). 20203100 10.1210/me.2009-0507 PMC2870936

[b25] Wang Y., Kumar N., Crumbley C., Griffin P. R. & Burris T. P. A second class of nuclear receptors for oxysterols: Regulation of RORalpha and RORgamma activity by 24S-hydroxycholesterol (cerebrosterol). Biochim. Biophys. Acta 1801, 917–923 (2010). 20211758 10.1016/j.bbalip.2010.02.012 PMC2886165

[b26] Fauber B. P. *et al.* Structure-based design of substituted hexafluoroisopropanol-arylsulfonamides as modulators of RORc. Bioorg. Med. Chem. Lett. 23, 6604–6609 (2013). 24239186 10.1016/j.bmcl.2013.10.054

[b27] Kumar N. *et al.* The benzenesulfoamide T0901317 [N-(2,2,2-trifluoroethyl)-N-[4-[2,2,2-trifluoro-1-hydroxy-1-(trifluoromethyl)ethyl]phenyl]-benzenesulfonamide] is a novel retinoic acid receptor-related orphan receptor-α/γ inverse agonist. Mol. Pharmacol. 77, 228–236 (2010). 19887649 10.1124/mol.109.060905 PMC2812071

[b28] Ichiyama K. *et al.* Foxp3 inhibits RORγt-mediated IL-17A mRNA transcription through direct interaction with RORγt. J. Biol. Chem. 283, 17003–17008 (2008). 18434325 10.1074/jbc.M801286200

[b29] Fauber B. P. *et al.* Discovery of imidazo[1,5-a]pyridines and -pyrimidines as potent and selective RORc inverse agonists. Bioorg. Med. Chem. Lett. 25, 2907–2912 (2015). 26048793 10.1016/j.bmcl.2015.05.055

[b30] Chaikuad A. *et al.* A unique inhibitor binding site in ERK1/2 is associated with slow binding kinetics. Nat. Chem. Biol. 10, 853–860 (2014). 25195011 10.1038/nchembio.1629 PMC4687050

[b31] Yang T. *et al.* Discovery of Tertiary Amine and Indole Derivatives as Potent RORγt Inverse Agonists. ACS Med. Chem. Lett. 5, 65–68 (2014). 24900774 10.1021/ml4003875 PMC4027777

[b32] Stehlin C. *et al.* X-ray structure of the orphan nuclear receptor RORbeta ligand-binding domain in the active conformation. EMBO J. 20, 5822–5831 (2001). 11689423 10.1093/emboj/20.21.5822 PMC125710

[b33] Studier F. W. Protein production by auto-induction in high density shaking cultures. Protein Expr. Purif. 41, 207–234 (2005). 15915565 10.1016/j.pep.2005.01.016

[b34] Weeks S. D., Drinker M. & Loll P. J. Ligation Independent Cloning Vectors for Expression of SUMO Fusions. Protein Expr. Purif. 53, 40–50 (2007). 17251035 10.1016/j.pep.2006.12.006 PMC1892228

[b35] Battye T. G. G., Kontogiannis L., Johnson O., Powell H. R. & Leslie A. G. W. iMOSFLM: a new graphical interface for diffraction-image processing with MOSFLM. Acta Crystallogr. D Biol. Crystallogr. 67, 271–281 (2011). 21460445 10.1107/S0907444910048675 PMC3069742

[b36] Evans P. R. & Murshudov G. N. How good are my data and what is the resolution? Acta Crystallogr. D Biol. Crystallogr. 69, 1204–1214 (2013). 23793146 10.1107/S0907444913000061 PMC3689523

[b37] Winn M. D. *et al.* Overview of the CCP4 suite and current developments. Acta Crystallogr. D Biol. Crystallogr. 67, 235–242 (2011). 21460441 10.1107/S0907444910045749 PMC3069738

[b38] McCoy A. J. *et al.* Phaser crystallographic software. J. Appl. Crystallogr. 40, 658–674 (2007). 19461840 10.1107/S0021889807021206 PMC2483472

[b39] Bruno I. J. *et al.* Retrieval of crystallographically-derived molecular geometry information. J. Chem. Inf. Comput. Sci. 44, 2133–2144 (2004). 15554684 10.1021/ci049780b

[b40] Emsley P., Lohkamp B., Scott W. G. & Cowtan K. Features and development of Coot. Acta Crystallogr. D Biol. Crystallogr. 66, 486–501 (2010). 20383002 10.1107/S0907444910007493 PMC2852313

[b41] Adams P. D. *et al.* *PHENIX*: a comprehensive Python-based system for macromolecular structure solution. Acta Crystallogr. D Biol. Crystallogr. 66, 213–221 (2010). 20124702 10.1107/S0907444909052925 PMC2815670

[b42] Afonine P. V. *et al.* Towards automated crystallographic structure refinement with phenix.refine. Acta Crystallogr. D Biol. Crystallogr. 68, 352–367 (2012). 22505256 10.1107/S0907444912001308 PMC3322595

[b43] Chen V. B. *et al.* MolProbity: all-atom structure validation for macromolecular crystallography. Acta Crystallogr. D Biol. Crystallogr. 66, 12–21 (2010). 20057044 10.1107/S0907444909042073 PMC2803126

[b44] Laskowski R. A. & Swindells M. B. LigPlot+: multiple ligand-protein interaction diagrams for drug discovery. J. Chem. Inf. Model. 51, 2778–2786 (2011). 21919503 10.1021/ci200227u

[b45] Ivanov I. I. *et al.* The orphan nuclear receptor RORγt directs the differentiation program of proinflammatory IL-17+ T helper cells. Cell 126, 1121–1133 (2006). 16990136 10.1016/j.cell.2006.07.035

