# Supplementary material for: Identification of an allosteric binding site for RORγt inhibition
Source: Nat Commun. 2015 Dec 7;6:8833. doi: 10.1038/ncomms9833 (PMC4686831; doi:10.1038/ncomms9833)
Supplement: Supplementary Information — Supplementary Figures 1-6, Supplementary Table 1, Supplementary Note 1, Supplementary Methods and Supplementary Reference [file ncomms9833-s1.pdf]

Supplementary Figures.

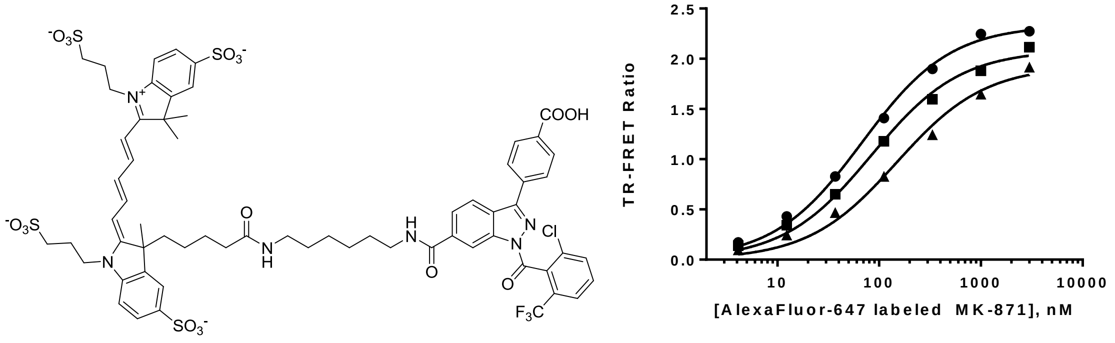

Supplementary Figure 1: Structure and biochemical activity of the AlexaFluor 647 labeled MRL-871 allosteric probe. Left) Molecular structure of the allosteric probe. Right) Titration of allosteric probe molecule at 5 nM (circles), 2.5 nM (squares), 1.25 nM (triangles) to RORγt-LBD in a TR-FRET assay.

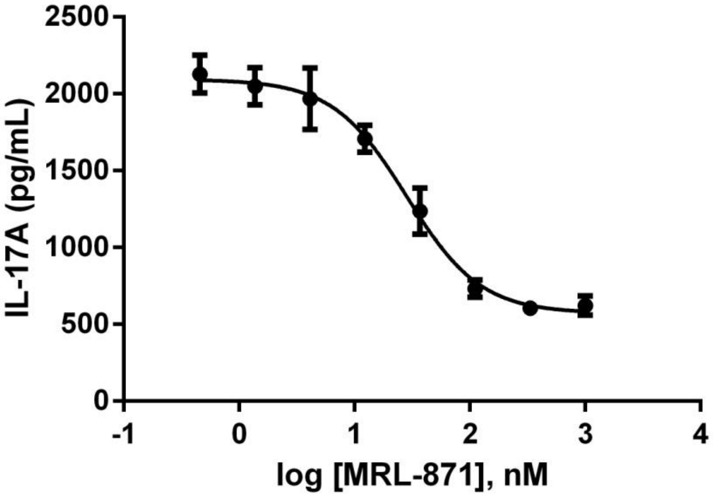

Supplementary Figure 2: Dose response curve for IL-17A production. IL-17A values were determined versus a standard curve of recombinant IL-17A, following the experimental setting as described under PBMC Th17 polarization and IL-17 production assay.

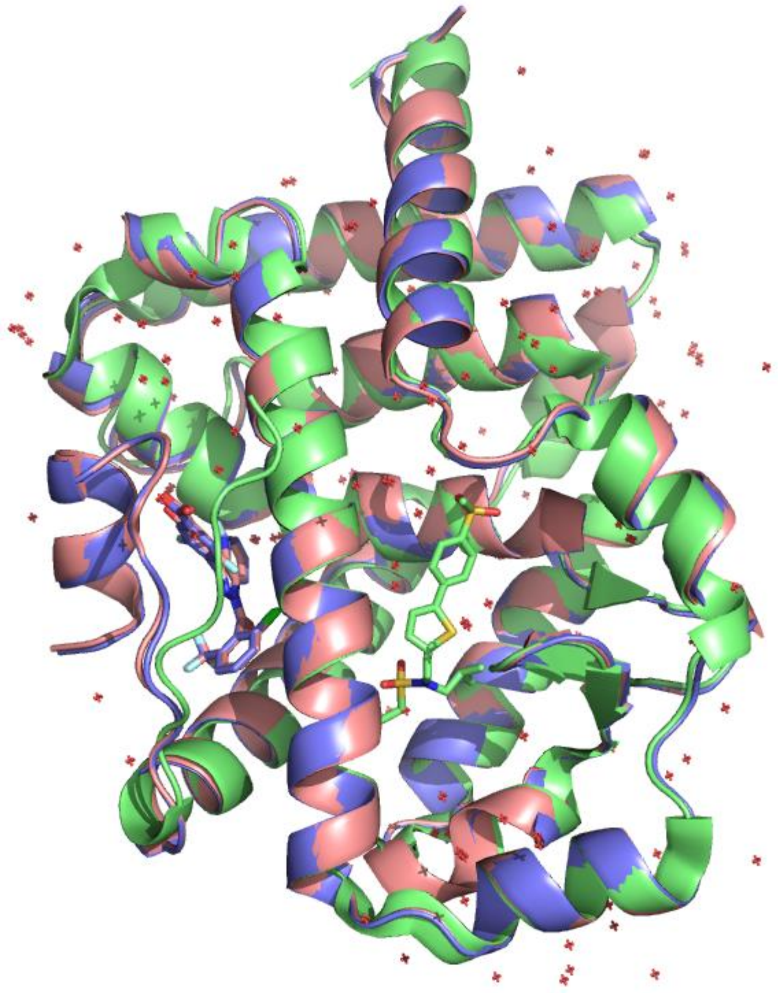

Supplementary Figure 3: Overall structural comparison of human RORγt in complex with MRL-299 (Blue), MRL-367 (Pink), and a tertiary sulfonamide ROR inverse agonist (Green, 4QM0).

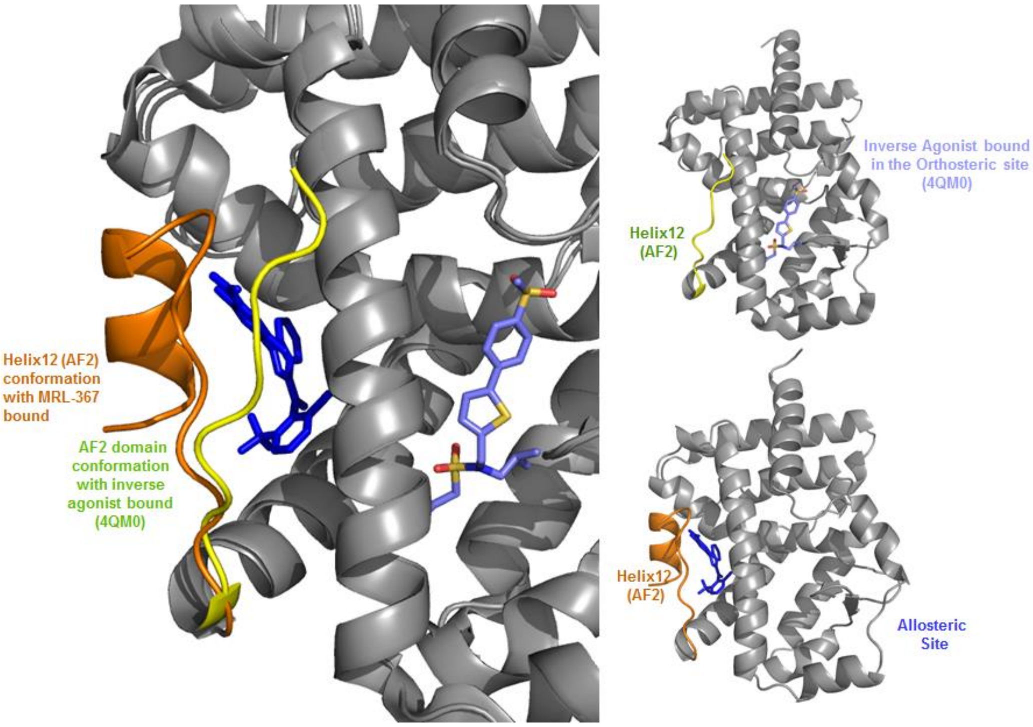

Supplementary Figure 4: Helix 12 conformational changes upon compound binding. The presence of the MRL-367 induces a conformational change extending the Helix12/AF2 domain into the co-activator binding region. This in turn forms the binding pocket for the ligand. The inverse agonist in the orthosteric site induces a disordered Helix-12/AF2 domain.

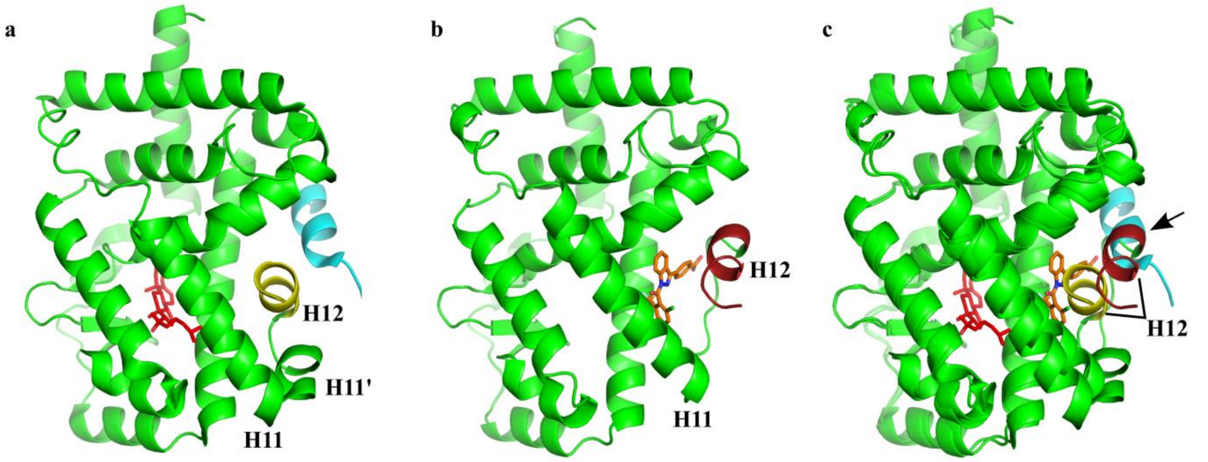

Supplementary Figure 5: Allosteric mechanism of RORγt with MRL-871. a) Agonistic conformation of RORγt published by Martynowski and coworkers (PDB entry 3KYT) showing the position of the 20α hydroxycholesterol (red) in the ligand binding site, helix 12 of the LBD (yellow) and the bound LXXLL cofactor (blue). b) Antagonistic conformation of RORγt, with **MRL-871** (orange) making contact with helix 12 (brown) at the allosteric site in the RORγt LBD. c) Superposition of the agonistic and allosteric antagonistic RORγt conformations, with colors corresponding to those in panels a and b. The position of helix 12 has shifted to the position of the co-factor peptide, demonstrating the allosteric inverse agonistic mechanism.

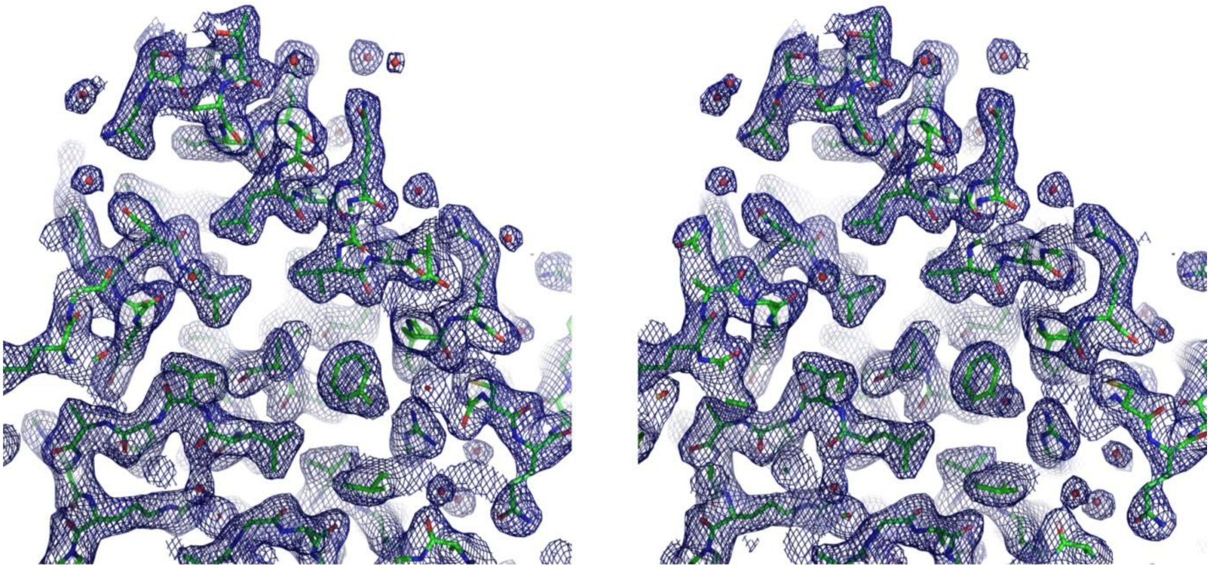

Supplementary Figure 6: Stereo image of a portion of the electron density map (2Fo-Fc, contoured at 1 σ) of co-crystal structure of RORγt with **MRL-871 (**4YPQ).

Supplementary Table 1

Table S1 – Selectivity of MRL-299 across a panel of receptor transactivation assays*

Receptor	Agonist EC50 (nM)	Antagonist IC50 (nM)
AR	> 30000	12000
AhR	>30000	n.d.
CAR1		16650
CAR2	> 30000	n.d.
CAR3	> 30000	n.d.
ERa	> 30000	> 30000
ERb	> 30000	10800
FXR	> 30000	13571
GR	> 30000	18500
LXRa	> 30000	23200
LXRb	> 30000	21500
MR	> 30000	20026
PPARa	> 30000	19100
PPARd	> 30000	28000
PPARg	686	517 **
PR	> 30000	>30000
PXR	4624 (63% of rifampicin max)***	>30000
RARa	> 30000	ND
RARb	> 30000	12253
RARg	364 (20% of ATRA max)***	3108
RORg	n.d.	2.9
RXRa	> 30000	7419
RXRb	> 30000	28000
RXRg	> 30000	n.d.
TRa	> 30000	4490
TRb	> 30000	5731
VDR	> 30000	> 30000

* Receptor Reporter Assay Selectivity Panel- Assays were performed by Indigo Bioscience (State College, PA) according to their established protocols. The AR, ER, GR, MR, and PR assays utilize full length receptor constructs whereas all others are chimeric with the GAL4 DNA binding domain.

** PPARg antagonism exhibited a bell-shaped dose response curve

*** MRL-299 showed partial agonist activity against PXR and RARg relative to rifampicin and ATRA controls.

n.d. = not determined

Supplementary Note 1

Selectivity of MRL-299 against a panel of nuclear receptors. MRL-299 was tested against a commercially available panel of cell-based nuclear receptor reporter assays (Table S1). The compound was >100-fold specific for RORγ vs. other tested nuclear receptors. Although MRL-299 is highly selective for RORγt, modest activity against PPARγ warranted additional experiments in physiologically relevant PPARγ assays. Human mesenchymal stem cells (MSCs) are dependent on PPARγ for differentiation to adipocytes and thus were cultured under adipogenic conditions in the presence of MRL-299 or the PPARγ antagonist GW9662. While GW9662 inhibited adipogenesis with an IC50 of 5.4 μM, the IC50 of MRL-299 was ~50 μM (data not shown). In a similar experiment, GW9662 inhibited adiponectin gene expression with an IC50 of 1 μM whereas the MRL-299 IC50 was ~30 μM (data not shown). To assess the agonist potential of MRL-299, induction of CD36 and FABP4 gene expression in MSCs was assessed in comparison to the synthetic PPARγ agonist rosiglitazone. Rosiglitazone induced a 3-fold and 2-fold induction of each gene respectively, but no induction by MRL-299 was observed up to 30 μM (data not shown).

Supplementary Methods

Synthetic procedures

All the solvents employed were commercially available and used without purification unless stated otherwise. Water was purified using a Millipore purification train. All the reagents are commercially available and used without purification. All the NMR data were recorded on a Varian Gemini 400 MHz NMR or a Bruker Cryomagnet for NMR spectroscopy 400 MHz (400 MHz for ^{1}H-NMR and 100 MHz for ^{13}C-NMR). Proton experiments are reported in parts per million (ppm) downfield of TMS and were relative to the residual methanol (3.31 ppm) or chloroform (7.26 ppm). All ^{13}C spectra were reported in ppm relative to residual methanol (49.00 ppm) or chloroform (77 ppm) Analytical LC-MS was performed on a C4, Jupiter SuC4300A, 150x2.00 mm column with a gradient 5% to 100% acetonitrile in H_2O in 15 min. Silica column chromatography was performed manually using silica with particle size 60 – 200 µm. Preparative HP-LC was performed on a Gemini S4 110A 150x21.20 mm column using H_2O with 0.1% Formic Acid (F.A.) and acetonitrile with 0.1% F.A. Gradient: 40% to 60% acetonitrile in 20 minutes. Purity and exact mass of the compounds were determined using a High Resolution LC-MS system consisting of a Waters ACQUITY UPLC I-Class system coupled to a Xevo G2 Quadrupole Time of Flight (Q-tof). The system was comprised of a Binary Solvent Manager and a Sample Manager with Fixed-Loop (SM-FL). compounds were separated (0.3 mL min^{-1}) by the column (Polaris C18A reverse phase column 2.0 x 100 mm, Agilent) using a 15% to 75% acetonitrile gradient in water supplemented with 0.1% v/v formic acid before analysis in positive mode in the mass spectrometer. Synthesis was performed following and adapting procedures[1] by Merck & Co. Compound T0901317 was commercially obtained from Sigma Aldrich and used without further purification.

Preparation of 4-(1-(2-chloro-6-(trifluoromethyl)benzoyl)-1H-indazol-3-yl)benzoic acid (MRL-871)

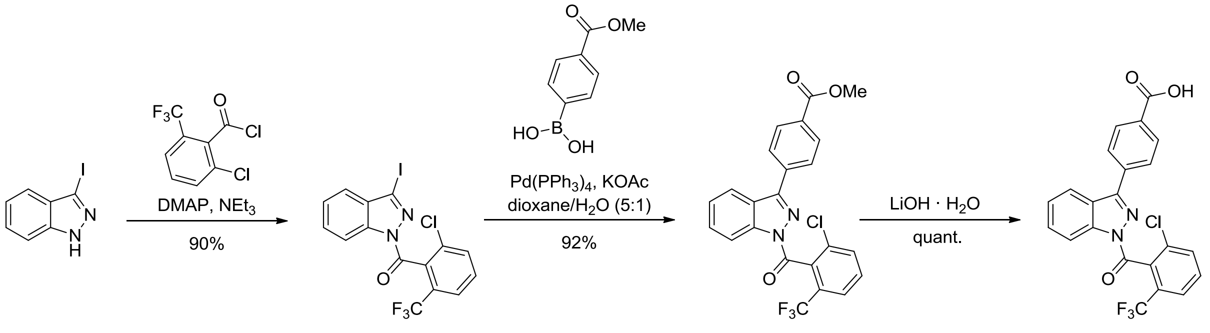

(2-chloro-6-(trifluoromethyl)phenyl)(3-iodo-1H-indazol-1-yl)methanone

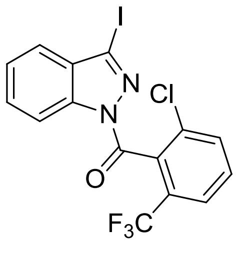

Thionyl chloride (1 mL, 13.8 mmol) was added dropwise to 2-chloro-6-(trifluoromethyl)benzoic acid (100 mg, 0.45 mmol) in an oven dried flask. The reaction mixture was stirred at 75 °C overnight. After removal of the thionyl chloride under reduced pressure, the benzoyl chloride was dissolved in dry CH_2Cl_2 (1 mL). To this solution 3-iodo-1H-indazole (108.5 mg, 0.45 mmol) and DMAP (54.4 mg, 0.46 mmol) were added. Finally, TEA (130 µl, 0.9 mmol) was added dropwise and the mixture was stirred at room temperature.

After 24 h the reaction mixture was diluted with H_2O (8 mL) and CH_2Cl_2 (8 mL). The layers were separated and the aqueous layer was washed with CH_2Cl_2 (2 x 8 mL). The combined organic layers were washed with brine, dried over Na_2SO_4, filtered and the solvent evaporated. The crude material was purified via silica column chromatography eluting with pentane/EtOAc 5% to 8% (v/v) to yield 183.3 mg (2-chloro-6-(trifluoromethyl)phenyl)(3-iodo-1H-indazol-1-yl)methanone as white solid, 90.4%. LC-MS (ESI): calc. for $C_{15}H_7ClF_3IN_2O$ [M+H]: 451.6 observed 451.1, LC, Rt=7.17 min. ^{1}H-NMR (400 MHz, $CDCl_3$): δ (ppm) 8.53 (d, J = 8.4 Hz, 1H), 7.74-7.67 (m, 3H), 7.61-7.49 (m, 3H); ^{13}C-NMR (100 MHz, $CDCl_3$): δ 164.02, 138.95, 133.12, 132.88, 131.86, 131.10, 131.03, 126.09, 124.94, 124.89, 124.55, 124.35, 122.20, 115.61, 105.63.

methyl 4-(1-(2-chloro-6-(trifluoromethyl)benzoyl)-1H-indazol-3-yl)benzoate

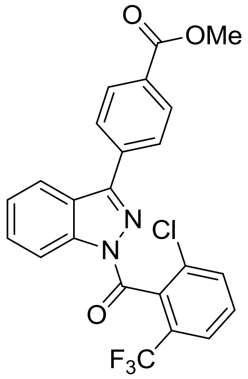

(2-chloro-6-(trifluoromethyl)phenyl)(3-iodo-1H-indazol-1-yl)methanone (115.7 mg, 0.26 mmol), (4-(methoxycarbonyl)phenyl)boronic acid (71.9 mg, 0.4 mmol), $Pd(PPh_3)_4$ (19.4 mg, 26.5 µmol) and CH_3CO_2K (80.5 mg, 0.82 mmol) were dissolved in dioxane/H_2O (5:1 v/v, 5 mL) in a schlenk tube under argon. The reaction was stirred at 90 ºC. After 3 hours the mixture was allowed to cool to room temperature and diluted with CH_2Cl_2 (25 mL) and H_2O (25 mL). The organic layer was washed with brine, dried over Na_2SO_4, filtered and concentrated *in vacuo*. The obtained crude material was purified via silica column chromatography (eluent: pentane/EtOAc, 4% to 10% v/v) to yield compound methyl 4-(1-(2-chloro-6-(trifluoromethyl)benzoyl)-1H-indazol-3-yl)benzoate as a pale yellow solid, 109 mg, 92%. LC-MS (ESI): calc. for $C_{23}H_{14}ClF_3N_2O_3$ [M+H]: 459.8 observed 459.3 LC, Rt=7.53 min. ^{1}H-NMR (400 MHz, $CDCl_3$): δ (ppm) 8.69 (d, J = 8.4 Hz, 1H), 8.13 (d, J = 8.4 Hz, 2H), 8.02 (d, J = 8.0 Hz, 1H), 7.90 (d, J = 8.4 Hz, 2H), 7.74-7.69 (m, 3H), 7.62-7.52 (m, 2H), 3.95 (s, 3H); ^{13}C-NMR (100 MHz, $CDCl_3$): δ 166.71, 164.91, 150.54, 140.61, 135.84, 133.05, 132.86, 131.17, 130.87, 130.19, 130.14, 128.31, 126.12, 125.13, 124.92, 124.88, 121.44, 116.14, 52.46.

4-(1-(2-chloro-6-(trifluoromethyl)benzoyl)-1H-indazol-3-yl)benzoic acid (MRL-871)

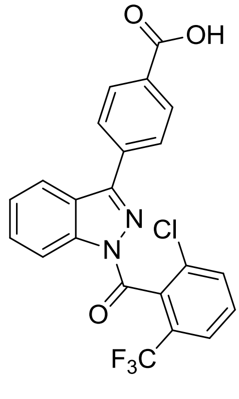

LiOH · H_2O (31.2 mg, 0.74 mmol) was added to a solution of methyl 4-(1-(2-chloro-6-(trifluoromethyl)benzoyl)-1H-indazol-3-yl)benzoate (107.1 mg, 0.23 mmol) in THF/H_2O (0.9 mL). The reaction was stirred at room temperature. After 24 h the reaction was diluted with H_2O, neutralized with acetic acid (~pH 4) and extracted with CH_2Cl_2. The combined organic layers were washed with brine, dried over Na_2SO_4, filtered and concentrated *in vacuo*. The product was purified via preparative LCMS. LC-MS (ESI): calc. for $C_{22}H_{12}ClF_3N_2O_3$ [M+H]: 445.8 observed 445.3 LC, Rt=6.83 min. ^{1}H-NMR (400 MHz, CD_3OD): δ (ppm) 8.63 (d, J = 8.4 Hz, 1H), 8.20 (d, J = 8.0 Hz, 1H), 8.16 (d, J = 8.4 Hz, 2H), 7.97 (d, J = 8.4 Hz, 2H), 7.89-7.75 (m, 4H), 7.64 (t, J = 7.6 1H); ^{13}C-NMR (100 MHz, CD_3OD): δ 169.10, 162.83, 151.81, 141.68, 136.61, 134.43, 133.63, 133.18, 132.69, 131.39, 131.33, 129.11, 127.45, 126.08, 122.91, 116.45. ^{19}F-NMR (376 MHz, CD_3OD): δ -61.15. HRMS (m/z): $[M + H]^+$ calcd for $C_{22}H_{12}ClF_3N_2O_3$, 445.0566, found 445.0577. MS/MS (m/z): most abundant peaks: 364.0823, 209.9834, 208.9799, 206.9828 and 178.9877.

Preparation of 4-(1-(2-(trifluoromethyl)benzoyl)-1H-indazol-3-yl)benzoic acid (MRL-058)

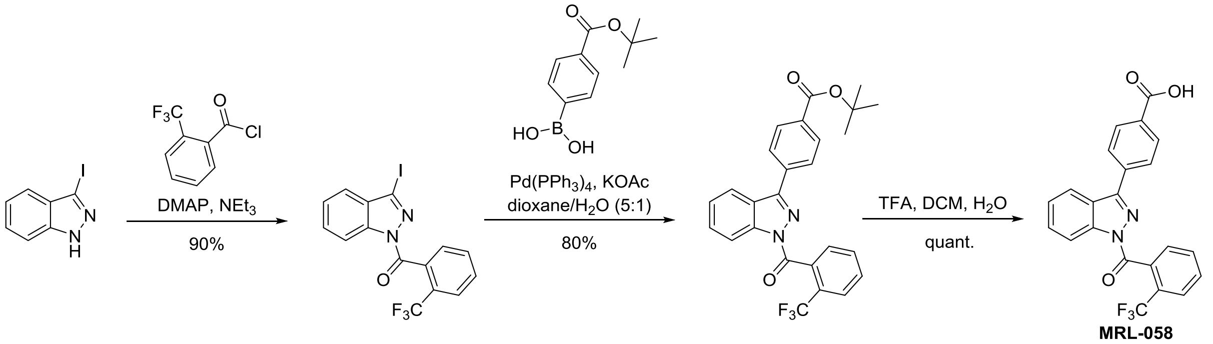

(3-iodo-1H-indazol-1-yl)(2-(trifluoromethyl)phenyl)methanone

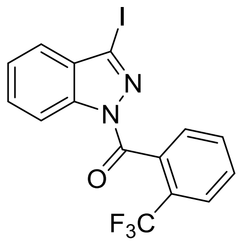

Thionyl chloride (1 mL, 13.8 mmol) was added dropwise to 2-(trifluoromethyl)benzoic acid (85.5 mg, 0.46 mmol) in an oven dried flask. The reaction mixture was stirred overnight at 75 °C. After removal of the thionyl chloride under reduced pressure, the benzoyl chloride was dissolved in dry CH_2Cl_2 (1 mL). To this solution 3-iodo-1H-indazole (113 mg, 0.46 mmol), DMAP (56 mg, 0.46 mmol) were added. Finally, TEA (130 µl, 0.9 mmol) was added dropwise and the mixture was stirred at room temperature.

After 24 h the reaction mixture was separated between H_2O (10 mL) and CH_2Cl_2 (10 mL). The aqueous layer was extracted with CH_2Cl_2 (2 x 10 mL). The combined organic layers were washed with brine, dried over Na_2SO_4, filtered and concentrated *in vacuo*. The crude material was purified via silica column chromatography eluting with pentane/EtOAc 5% (v/v) to yield 146 mg (3-iodo-1H-indazol-1-yl)(2-(trifluoromethyl)phenyl)methanone as white solid, 90%. GC-MS (EI) expected for $C_{15}H_8F_3IN_2O$: 415.96, observed [M$^{+\bullet}$]: 416 with fragments: 347, 173, 145; ^{1}H-NMR (400 MHz, $CDCl_3$): δ (ppm) 8.53 (d, J = 8.4 Hz, 1H), 7.81-7.79 (m, 1H), 7.73-7.66 (m, 3H), 7.60-7.84 (m, 3H); ^{13}C-NMR (100 MHz, $CDCl_3$): δ 166.86, 139.44, 132.88, 131.53, 131.02, 130.98, 130.59, 129.16, 126.75, 126.71, 125.92, 124.98, 122.14, 115.75, 105.07.

tert-butyl 4-(1-benzoyl-1H-indazol-3-yl)benzoate

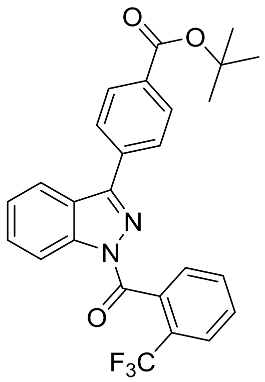

In an oven dried schlenk tube were weighed: (4-(tert-butoxycarbonyl)phenyl)boronic acid (117 mg, 0.53 mmol), KOAc (106 mg, 1.08 mmol) and Pd(dppf)Cl_2 (28.4 mg, 39 µmol). Finally (3-iodo-1H-indazol-1-yl)(2-(trifluoromethyl)phenyl)methanone (144 mg, 0.35 mmol) in dioxane/H_2O (5:1 v/v, 5.2 mL) was added and the reaction was stirred for 3.5 h at 90 °C. The reaction mixture was allowed to cool to room temperature and was then separated between CH_2Cl_2 and H_2O. The aqueous layer was extracted with CH_2Cl_2 twice. The combined organic layers were washed with brine, dried over Na_2SO_4, filtered and evaporated. The product was purified via silica column chromatography eluting with 45% pentane in CH_2Cl_2 (R_f = 0.35) to yield tert-butyl 4-(1-benzoyl-1H-indazol-3-yl)benzoate as an off-white solid. Yield: 80%, 129 mg, 0.28 mmol. LC-MS (ESI): calc. for $C_{26}H_{21}F_3N_2O_3$ [M+H]: 467.16

observed 467.17, LC, Rt=9.67 min. ^{1}H-NMR (200 MHz, $CDCl_3$): δ (ppm) 8.67 (d, J = 8.4 Hz, 1H), 8.12-7.99 (m, 3H), 7.94-7.63 (m, 7H), 7.61-7.43 (m, 1H), 1.60 (s, 9H); ^{13}C-NMR (50 MHz, $CDCl_3$): δ 167.92, 165.36, 150.09, 141.06, 135.39, 133.56, 132.95, 131.54, 130.39, 130.03, 129.03, 128.05, 126.68, 126.59, 125.90, 125.01, 121.42, 116.24, 81.52, 28.32; ^{19}F-NMR (188 MHz, $CDCl_3$) δ -59.29.

4-(1-(2-(trifluoromethyl)benzoyl)-1H-indazol-3-yl)benzoic acid (MRL-058)

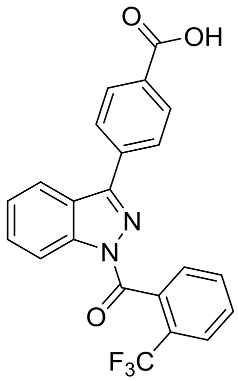

tert-butyl 4-(1-(2-(trifluoromethyl)benzoyl)-1H-indazol-3-yl)benzoate (128 mg, 0.27 mmol) was disolved in a mixture of CH_2Cl_2/TFA/H_2O (6.5/3/0.5, 3 mL). The reaction was stirred at room temperature. After 2.5 h the solvent was evaporated and the product was purified via preparative LCMS.

LC-MS (ESI): calc. for $C_{22}H_{13}F_3N_2O_3$ [M+H]: 411.10 observed 411.17, LC, Rt=7.58 min. ^{1}H-NMR (400 MHz, $CDCl_3$ with 10% CD_3OD) δ 8.65 (d, *J* = 8.4 Hz, 1H), 8.16 (d, *J* = 8.1 Hz, 2H), 8.07 (d, *J* = 8.1 Hz, 1H), 7.94 (d, *J* = 8.0 Hz, 2H), 7.86 – 7.82 (m, 1H), 7.77 – 7.64 (m, 4H), 7.56 (m, 1H); ^{13}C-NMR (100 MHz, $CDCl_3$ with 10% CD_3OD): δ 168.19, 167.95, 150.05, 140.80, 133.17, 133.15, 130.28, 130.17, 129.87, 128.72, 127.93, 127.66, 126.39, 126.35, 125.80, 124.89, 124.77, 122.17, 121.28; ^{19}F-NMR (188 MHz, $CDCl_3$ with 10% CD_3OD) δ -55.63; HRMS (m/z): $[M + H]^+$ calcd for $C_{22}H_{13}F_3N_2O_3$, 411.0956, found 411.0957. MS/MS (m/z): most abundant peaks: 391.0890, 174.0244, 173.0212 and 145.0264.

Preparation of 4-(1-benzoyl-1H-indazol-3-yl)benzoic acid (MRL-003)

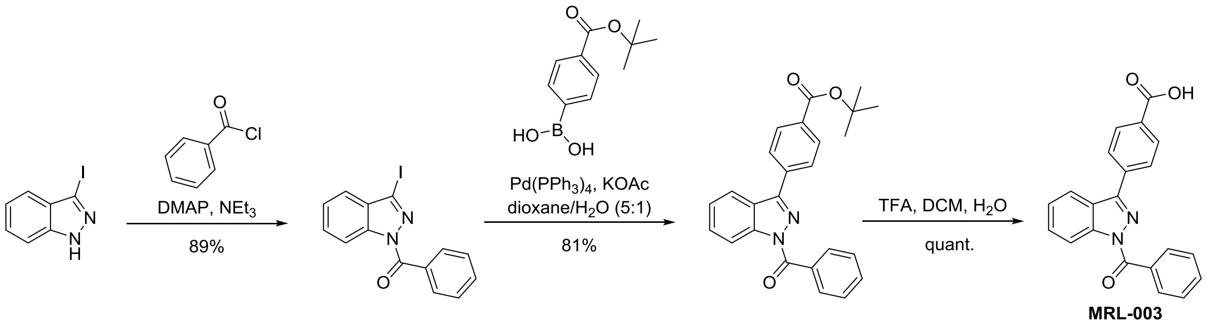

(3-iodo-1H-indazol-1-yl)(phenyl)methanone

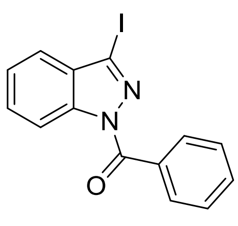

3-iodo-1H-indazole (100 mg, 0.42 mmol) and DMAP (53 mg, 0.44 mmol) were dissolved in CH_2Cl_2 (1.5 mL). Benzoyl chloride (48 µL, 0.42 mmol) and NEt_3 (114 µL, 0.82 mmol) were added and the reaction was stirred for 24 h at room temperature. The reaction mixture was then separated between CH_2Cl_2 and H_2O. The aqueous layer was extracted with CH_2Cl_2 twice. The combined organic layers were washed with brine, dried over Na_2SO_4, filtered and evaporated. The product was purified via silica column chromatography to yield (3-iodo-1H-indazol-1-yl)(phenyl)methanone as a pale yellow solid. 130 mg, 0.37 mmol, 89%. GC-MS (EI) expected for $C_{14}H_9IN_2O$: 347.98, observed $[M^{+\bullet}]$: 348 with fragments: 105, 77; ^{1}H-NMR (400 MHz, $CDCl_3$): δ (ppm) 8.53 (d, *J* = 8.4 Hz, 1H), 8.11 - 8.09 (m, 2H), 7.69 (ddd, *J* = 8.4, 7.0, 1.3 Hz,

1H), 7.64 - 7.59 (m, 1H), 7.57 - 7.46 (m, 4H); ^{13}C-NMR (100 MHz, $CDCl_3$): 167.43, 140.24, 132.73, 132.69, 131.45, 130.72, 130.51, 128.22, 125.47, 121.95, 116.03, 104.01.

tert-butyl 4-(1-benzoyl-1H-indazol-3-yl)benzoate

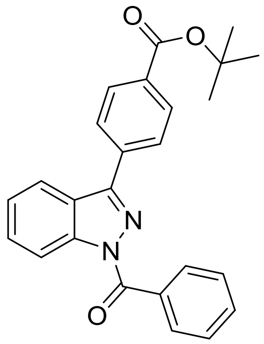

In an oven dried schlenk tube were weighed: (4-(tert-butoxycarbonyl)phenyl)boronic acid (123 mg, 0.55 mmol), KOAc (107 mg, 1.09 mmol) and Pd(dppf)Cl_2 (26 mg, 36 µmol). Finally (3-iodo-1H-indazol-1-yl)(phenyl)methanone (126 mg, 0.36 mmol) in dioxane/H_2O (5:1 v/v, 5.3 mL) was added and the reaction was stirred for 3 h at 90 °C. The reaction mixture was allowed to cool to room temperature and was then separated between CH_2Cl_2 and H_2O. The aqueous layer was extracted with CH_2Cl_2 twice. The combined organic layers were washed with brine, dried over Na_2SO_4, filtered and evaporated. The product was purified via silica column chromatography eluting with 40% v/v in CH_2Cl_2 (R_f = 0.25) to yield tert-butyl 4-(1-benzoyl-1H-indazol-3-yl)benzoate as a pale yellow paste. LC-MS (ESI): calc. for $C_{25}H_{22}N_2O_3$ [M+H]: 399.46 observed 399.08, LC, Rt=9.85 min. ^{1}H-NMR (400 MHz, $CDCl_3$): δ (ppm) 8.67 (d, J = 8.4 Hz, 1H), 8.22 – 8.11 (m, 4H), 8.07 – 8.00 (m, 3H), 7.70 – 7.59 (m, 2H), 7.57 – 7.46 (m, 3H), 1.63 (s, 9H); ^{13}C-NMR (50 MHz, $CDCl_3$) δ 168.36, 165.41, 149.38, 141.88, 135.80, 133.29, 132.84, 132.49, 131.51, 130.11, 129.70, 128.09, 128.03, 127.17, 125.46, 124.48, 121.20, 116.52, 81.52, 28.34.

4-(1-benzoyl-1H-indazol-3-yl)benzoic acid (MRL-003)

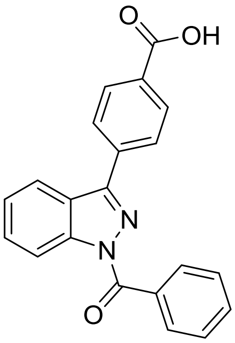

tert-butyl 4-(1-benzoyl-1H-indazol-3-yl)benzoate (102 mg, 0.26 mmol) was was disolved in a mixture of CH_2Cl_2/TFA/H_2O (6.5/3/0.5, 2.5 mL). The reaction was stirred at room temperature. After 2.5 h the solvent was evaporated and the product was purified via preparative LCMS. LC-MS (ESI): calc. for $C_{21}H_{14}N_2O_3$ [M+H]: 343.36 observed 343.08, LC, Rt=7.53 min. ^{1}H-NMR (400 MHz, $CDCl_3$ with 10% CD_3OD) δ (ppm) 8.65 (d, J = 8.5 Hz, 1H), 8.26 – 8.14 (m, 4H), 8.13 – 8.05 (m, 3H), 7.74 – 7.63 (m, 2H), 7.60 – 7.53 (m, 3H). ^{13}C-NMR (100 MHz, $CDCl_3$ with 10% CD_3OD) δ 168.55, 168.47, 149.44, 141.66, 135.88, 133.01, 132.39, 131.18, 130.31, 129.62, 127.96, 127.93, 125.41, 124.32, 121.11, 116.19. HRMS (m/z): $[M + H]^+$ calcd for $C_{21}H_{16}N_2O_3$, 343.1083, found 343.1078. MS/MS (m/z): most abundant peaks: 399.0266, 325.0970, 105.0337 and 77.0389.

Preparation of 4-(1-(2-chloro-6-(trifluoromethyl)benzoyl)-4-fluoro-1*H*-indazol-3-yl)-3-fluorobenzoate (MRL-299)

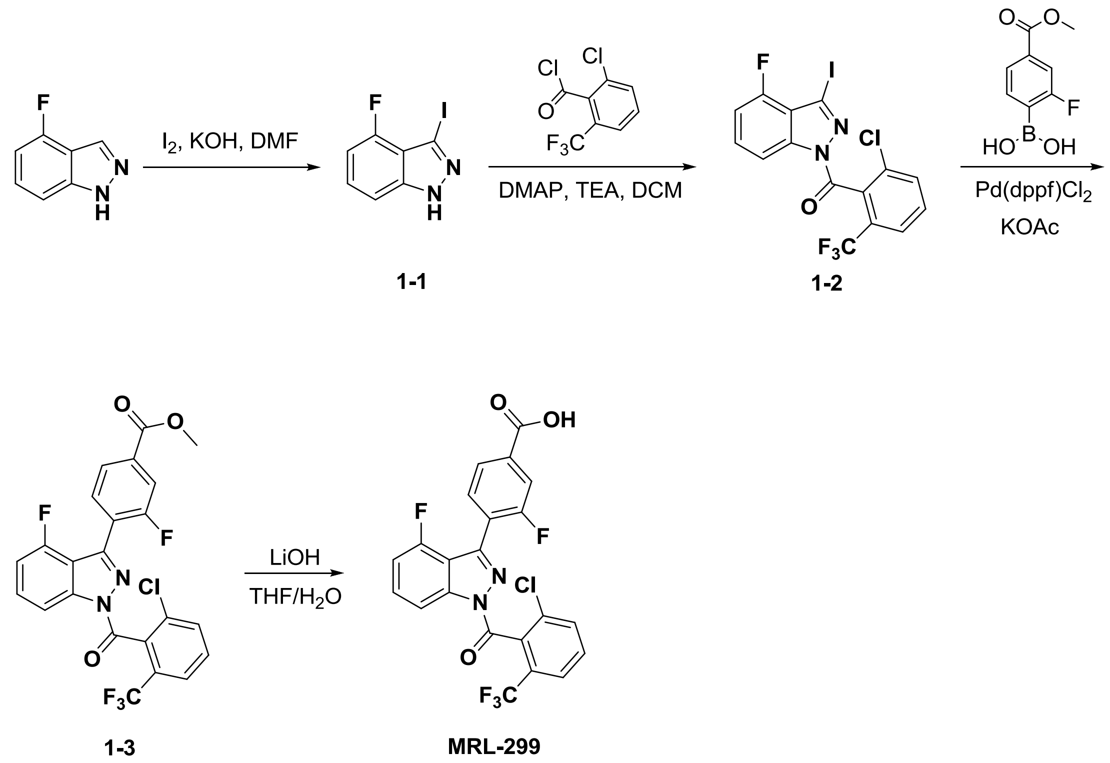

Step1: Preparation of 4-fluoro-3-iodo-1*H*-indazole (1-1). To a solution of 4-fluoroindazole (5.0 g, 37 mmol) in DMF (80 mL) at rt was added I_2 (18.6 g, 74 mmol) and KOH (7.73 g, 138 mmol) respectively. The reaction mixture was stirred at rt for 2 h, and TLC showed complete conversion. The reaction mixture was poured into aq. $NaHSO_3$ (10%, 200 mL) and extracted with EtOAc (200 mLx3). The combined organic layers were washed with H_2O and brine, dried over Na_2SO_4, and concentrated. The crude solid was washed with hexanes to give the desired product as a yellow solid (8.33 g, 86%). LCMS 263 (M+1).

Step2: Preparation of (2-chloro-6-(trifluoromethyl)phenyl)(4-fluoro-3-iodo-1*H*- indazol-1-yl)methanone (1-2). To a mixture of 4-fluoro-3-iodo-1*H*-indazole (5.24 g, 20 mmol), 2-chloro-6-(trifluoromethyl)benzoyl chloride (4.86 g, 20 mmol), DMAP (2.44 g, 20 mmol) and DCM (30 mL) was added TEA (5.8 mL, 40 mmol) drop wise. After the addition, the reaction mixture was kept stirring at rt for 14 h. The mixture was diluted with H_2O and extracted with DCM. The combined organics were washed with H_2O and brine, dried over Na_2SO_4, and concentrated. The residue was purified by silica gel flash chromatography (2-10% EtOAc/hexanes) to give the desired (7.8 g, 83%). LCMS 469 (M+1).

Step3: Preparation of methyl 4-(1-(2-chloro-6-(trifluoromethyl)benzoyl)-4- fluoro-1*H*-indazol-3-yl)-3-fluorobenzoate (1-3). A mixture of **1-2** (300 mg, 0.64 mmol), 2-fluoro-4-(methoxycarbonyl)phenylboronic acid (190 mg, 0.96 mmol), Pd(dppf)Cl_2 (52.2 mg, 0.064 mmol) and KOAc (190 mg, 1.92mmol) in dioxane (10 mL) and H_2O (2 mL) was heated at 90 ^oC for 2 h under microwave. The reaction mixture was cooled down, diluted with H_2O and extracted with EtOAc. The combined organics were washed with brine, dried over Na_2SO_4,

concentrated. The crude residue was purified by silica gel flash chromatography (0-5% EtOAc/hexanes) to give the desired product as a yellow solid (180 mg). LCMS 495 (M+1).

Step 4: Preparation of 4-(1-(2-chloro-6-(trifluoromethyl)benzoyl)-4-fluoro-1*H*- indazol-3-yl)-3-fluorobenzoic acid (MRL-299). To a solution of methyl 4-(1-(2-chloro-6-(trifluoromethyl)benzoyl)-4-fluoro-1H-indazol-3-yl)-3-fluorobenzoate (**1-3)** (180 mg, 0.36 mmol) in THF (5 ml) and H_2O (5 ml) was added LiOH (350 mg, 1.44 mmol). The reaction mixture was stirred at rt for 14 h, acidified with 2N HCl to PH = 3~4. The mixture was concentrated to give a white solid, which was rinsed with H_2O to furnish the desired product (160 mg). LCMS: 481 (M+1); 1H NMR (DMSO-d6, 600MHz) δ 13.6 (br s) 8.41 (1H, d, *J* = 8.5 Hz); 8.04 (1H, d, *J* = 8.2 Hz); 7.99 (1H, d, *J* = 8.2); 7.91 (2H, m); 7.86 (1H, t, *J* = 8.2 Hz); 7.85 (1H, dd, *J* = 10, 1.4 Hz); 7.70 (1H, t, *J* = 7.5 Hz); 7.47 (1H, dd, *J* = 10.2, 8.1 Hz); C13 NMR (DMSO-d6): 166.2 (d, *J* =2.5 Hz); 164.8; 160.1 (d, *J* = 250 Hz); 155.3 (d, *J* = 255 Hz); 144.4 (d, *J* = 3.1); 141.5 (d, *J* = 6.3 Hz); 135.4 (d, *J* = 6.9); 134.3; 133.9 (d, *J* = 8.1); 133.2; 132.3;; 132.1 (q, *J* = 2.1 Hz) ; 131.9; 128.1 (q, *J* = 32.1 Hz); 126.0 (q, *J* = 3.9 Hz); 126.0 (d, *J* = 3.9 Hz); 123.3 (q, *J* = 274 Hz); 122.8 (d, *J* = 14.8 Hz); 117.0 (d, *J* = 21.5 Hz); 115.0 (d, *J* = 19.6 Hz); 112.6 (d, *J* = 18.6 Hz); 111.8 (d, *J* = 3.9 Hz);

Preparation of 4-(1-(2-chloro-6-(trifluoromethyl)benzoyl)-1*H*-pyrazolo[4,3-b] pyridin-3-yl)-5-fluoro-2-hydroxybenzoate (MRL-367)

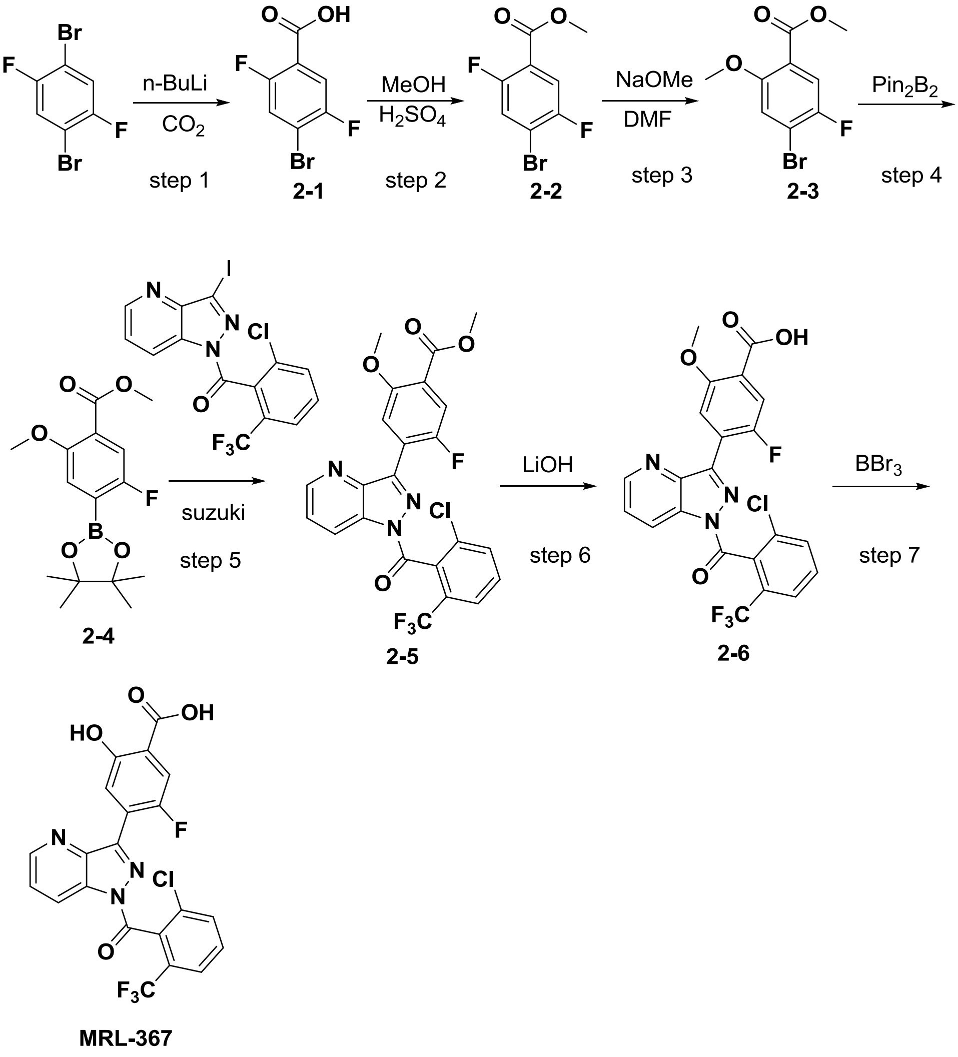

Step 1: Preparation of 4-bromo-2,5-difluorobenzoic acid (2-1). To a solution of 1,4-dibromo-2,5-difluorobenzene (2.7 g, 10 mmol) in Et_2O (60 ml) at -78 ^oC was added 2M ^nBuLi (6.0 ml, 12 mmol) drop wise, and the resultant mixture was kept stirring at -78 ^oC for 3 h. The resultant solution was poured into a flask containing excess dry ice, and the mixture rested at rt for 30 min. The mixture was diluted with H_2O and extracted with EtOAc. The aqueous layer was acidified by 2M HCl, and extracted with EtOAc. The combined organics were dried over Na_2SO_4, concentrated. The residue was purified by silica gel flash chromatography column (0-10% EtOAc/hexanes) to afford the desired product (1.51 g, 64%). LCMS: 237 (M+1).

Step 2. Preparation of methyl 4-bromo-2,5-difluorobenzoate (2-2). To a solution of 4-bromo-2,5-difluorobenzoic acid **2-1** (1.82 g, 7.73 mmol) in CH_3OH (50 ml) was added concentrated sulfuric acid (2 ml) drop wise. The reaction mixture was heated at 85 ^oC for 30 h, cooled down, and concentrated. The residue was purified by silica gel flash chromatography (0-5% EtOAc/hexanes) to afford the desired product (1.51 g , 91%). LCMS : 251 (M+1).

Step 3. Preparation of methyl 4-bromo-5-fluoro-2-methoxybenzoate (2-3). To a solution of methyl 4-bromo-2,5-difluorobenzoate **2-2** (1.76 g, 7.0 mmol) in DMF (30 ml) was added CH_3ONa (0.456 g, 8.45 mmol). The reaction mixture was stirred at rt for 16 h. The mixture was diluted with EtOAc and H_2O. The organic layer was separated, dried over Na_2SO_4, and concentrated. The residue was purified by silica gel flash chromatography (0-5% EtOAc/hexanes) to give the desired product (1.24 g, 67%). LCMS: 263 (M+1).

Step 4. Preparation of 2 methyl 5-fluoro-2-methoxy-4-(4,4,5,5-tetramethyl -1,3,2-dioxaborolan-2-yl)benzoate (2-4). A mixture of methyl 4-bromo-5-fluoro-2-methoxybenzoate **2-3** (1.42 g, 5.42 mmol), 4,4,4',4',5,5,5',5'-octamethyl-2,2'-bi(1,3,2-dioxa-borolane) (2.07 g, 8.13 mmol), Pd(dppf)Cl_2 (22 mg, 0.027 mmol) and dioxane (50 ml) was degassed and stirred at 80°C for 4 h. The reaction mixture was cooled down, filtered through celite, and concentrated. The residue was purified by silica gel flash chromatography (0-20% EtOAc/hexanes) to afford the desired product (1.06 g, 63.2%). LCMS: 310 (M+1).

Step 5. Preparation of methyl 4-(1-(2-chloro-6-(trifluoromethyl)benzoyl) -1*H*-pyrazolo [4,3-b]pyridin-3-yl)-5-fluoro-2-methoxybenzoate (2-5). A mixture of 2 methyl 5-fluoro-2-methoxy-4-(4,4,5,5-tetramethyl -1,3,2-dioxa borolan-2-yl)benzoate **2-4** (3.1 g, 10 mmol), (2-chloro-6-(trifluoro methyl)phenyl) (3-iodo-1H-pyrazolo[4,3-b]pyridin-1-yl)methanone (4.51 g, 10 mmol, prepared by following a similar procedure for the synthesis of (2-chloro-6-(trifluoromethyl)phenyl)(4-fluoro-3-iodo-1*H*-indazol-1-yl)methanone **1-2**), Pd$(dppf)_2Cl_2$ (41.2 mg, 0.05 mmol), K_2CO_3 (4.14 g, 30 mmol), dioxane (100 ml) was degassed and stirred at 80 ^{o}C for 4 h. The resultant mixture was cooled down, filtered through celite, and concentrated. The residue was purified by silica gel flash chromatography (0-20% EtOAc/hexanes) to afford the desired product (3.29 g 65%). LCMS: 508 (M+1).

Step 6. Preparation of 4-(1-(2-chloro-6-(trifluoromethyl)benzoyl)-1*H*-pyrazolo [4,3-b]pyridin-3-yl)-5-fluoro-2-methoxybenzoic acid (2-6). To a solution of methyl 4-(1-(2-chloro-6-(trifluoromethyl)benzoyl)-1H-pyrazolo[4,3-b] pyridin-3-yl)-5-fluoro-2-methoxybenzoate **2-5** (3.29 g, 6.49 mmol) in THF (80 ml) and H_2O (40 ml) was added LiOH (817 mg, 19.5 mmol). The reaction mixture was stirred at rt for 16 h, diluted with H_2O and acidified with 2M HCl. The combined organics were dried over Na_2SO_4 and concentrated. The residue was purified by silica gel flash chromatography (0-50% EtOAc/hexanes) to afford the desired product (2.79 g, 88%). LCMS: 494 (M+1).

Step 7. Preparation of 4-(1-(2-chloro-6-(trifluoromethyl) benzoyl)-1*H*-pyrazolo [4,3-b] pyridin-3-yl)-5-fluoro-2-hydroxybenzoic acid (MRL-367). To a solution of 4-(1-(2-chloro-6-(trifluoromethyl)benzoyl)-1*H*-pyrazolo[4,3-b] pyridin-3-yl)-5-fluoro-2-methoxybenzoic acid **2-6** (1.5 g, 3.0 mmol) in DCM (50 ml) was added BBr_3 (2.0 ml). The mixture solution was stirred at rt for 2 h and quenched by the addition of MeOH (30 ml) drop wise. The mixture was neutralized with excess Et_3N, concentrated. The residue was purified by silica gel flash chromatography (0-20% EtOAc/hexanes) to afford the desired product (1.1g, 76%). LCMS: 480 (M+1); ^{1}H NMR (400 MHz, DMSO-d6) δ 8.98 (1H, d, *J* = 4.4Hz), 8.90 (1H, d, *J* = 8.8Hz), 8.64 (1H, m), 8.01 (2H, m), 7.88 (2H, m), 7.64 (1H, d, *J* = 10Hz). C13 NMR (DMSO-d6): 170.6 (d, *J* = 2.2 Hz); 165.0; 156.9 (d, *J* = 2.1 Hz); 152.3 (d, *J* = 246 Hz); 150.6; 145.8 (d, *J* = 4.3 Hz); 142.7; 134.3; 133.2; 133.0; 132.0; 131.6 (q, *J* = 2.2 Hz); 128.3 (q, *J* = 32 Hz); 126.0 (q, *J* = 4.2 Hz); 125.5; 123.8 (d, *J* = 14.9 Hz); 123.5; 123.4 (q, *J* = 275 Hz); 120.1 (d, *J* = 1.9 Hz); 117.4 (d, *J* = 23.9 Hz); 115.7 (d, *J* = 7.2 Hz).

Preparation of ethyl 4-(1-(2-chloro-6-(trifluoromethyl)benzoyl)-4-fluoro-1*H*- indazol-3-yl)cyclohex-3-enecarboxylate (MRL-673)

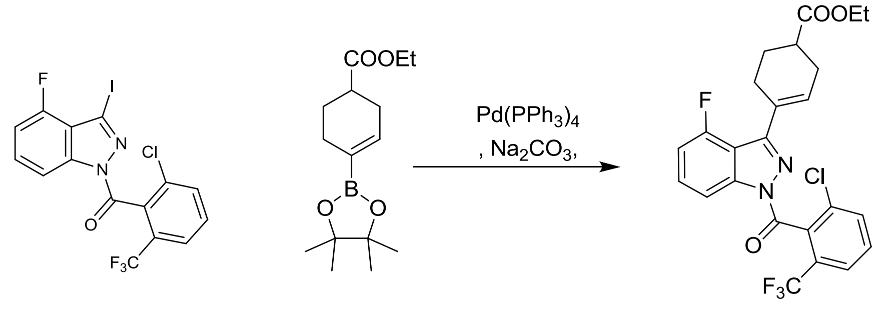

1-2 **3-1**

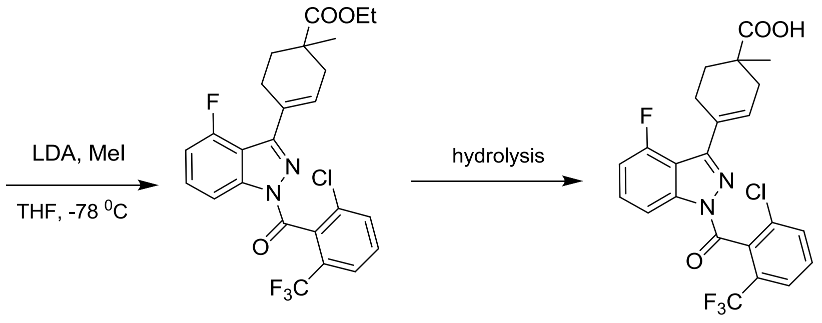

3-2 **MRL-673**

Step 1. Preparation of ethyl 4-(1-(2-chloro-6-(trifluoromethyl)benzoyl)-4-fluoro -1*H*-indazol-3-yl)cyclohex-3-enecarboxylate (3-1). To a mixture of (2-chloro-6-(trifluoromethyl)phenyl)(4-fluoro-3-iodo-1*H*-indazol-1-yl)methanone **1-2** (1 g, 2.1 mmol) in THF (40 mL) and H_2O (10mL) was added ethyl 4-(4,4,5,5-tetramethyl-1,3,2-dioxaborolan-2-yl)cyclohex-3-enecarboxylate (897 mg, 3.2 mmol) and Na_2CO_3 (667 mg, 6.3 mmol). The mixture was purged with nitrogen followed by the addition of Pd(dppf)Cl_2 (726 mg,0.63 mmol). The reaction mixture was heated at 80 ^oC for 10 h. The resulting mixture was cooled down, filtered through Celite. The filtrate was diluted with H_2O, and extracted with EtOAc. The combined organics were washed with brine, dried over Na_2SO_4 and concentrated. The crude residue was purified by silica gel flash chromatography (0-10% EtOAc/hexanes) to give the desired product (300 mg, 29%) as a brown oil. LCMS: 495 (M+1).

Step 2. Preparation of ethyl 4-(1-(2-chloro-6-(trifluoromethyl)benzoyl)-4-fluoro -1*H*-indazol-3-yl)-1-methylcyclohex-3-enecarboxylate (3-2). To a solution of ethyl 4-(1-(2-chloro-6-(trifluoromethyl)benzoyl)-4-fluoro-1*H*-indazol-3-yl)cyclohex-3-enecarboxylate **(3-1)** (200 mg, 0.40 mmol) in THF (1 mL) at -78 ^oC was added LDA (0.24 ml, 0.49 mmol) drop wise and the mixture was kept stirring for 15 min. To the resultant solution was added iodomethane (86 mg, 0.61 mmol). The mixture was stirred at -78 °C for 2 h, then warmed up to rt and stirred for 14 h. The reaction mixture was quenched with sat. NH_4Cl and extracted with EtOAc.

The combined organics were washed with brine, dried over anhydrous Na_2SO_4, and concentrated. The residue was purified by silica gel flash chromatography (0-10% EtOAc/hexanes) to give the desired product. (120 mg, yield: 56%) as yellow oil. LCMS: 509 (M+1).

Step 3: Preparation of 4-(1-(2-chloro-6-(trifluoromethyl)benzoyl)-4-fluoro -1*H*-indazol-3-yl)-1-methylcyclohex-3-enecarboxylic acid (MRL-673) A mixture of ethyl 4-(1-(2-chloro-6-(trifluoromethyl)benzoyl)-4-fluoro-1*H*-indazol-3-yl)-1 -methylcyclohex-3-enecarboxylate **(3-2)** (400 mg, 0.79 mmol) and NaOH (100 mg, 2.5 mmol) in EtOH (10 mL) was stirred at 40 ^{o}C for 12 h. The resulting mixture was concentrated, diluted with H_2O and extracted with EtOAc. The aqueous phase was then acidified with 2 M HCl. The precipitate solid was collected by filtration and purified by reverse HPLC (CH_3CN/H_2O and 0.075% TFA) to give the desired product. LCMS: 481 (M+1); H1 NMR (DMSO-d6): 12.2 (1H, br s), 8.30 (1H, d, *J* = 8.6 Hz); 7.97 (1H, d, *J* = 8.2 Hz); 7.91 (1H, d, *J* = 8.2 Hz); 7.80 (1H, t, *J* = 8.2 Hz); 7.76 (1H, td, *J* = 8.2, 5.4 Hz); 7.34 (1H, dd, *J* = 11.6, 8.4 Hz); 6.53 (1H, br s); 2.64 (1H, d, *J* = 18.6 Hz); 2.22 (2H, m) ; 2.05 (1H, dq, *J* = 18.6, 2.9 Hz); 1.86 (1H, dt, *J* = 13.8, 6.1 Hz); 1.53, dt, *J* = 12.8, 6.5 Hz); 1.12 (3H, s). C13 NMR (DMSO-d6): 178.5; 164.6; 155.2 (d, *J* = 254 Hz); 151.1 (d, *J* = 4.6 Hz); 141.8 (d, *J* = 7.5 Hz); 134.2; 133.1 (d, *J* = 8.6 Hz); 133.0 (d, *J* = 11.3 Hz); 132.8; 132.6 (q, *J* = 2.3 Hz); 131.8 (d, *J* = 3.3 Hz); 128.0 (d, *J* = 32.3 Hz); 127.5; 126.0 (q, *J* = 4.3 Hz); 126.0 (d, *J* = 3.9 Hz); 123.3(q, *J* = 274 Hz); 113.5 (d, *J* = 19.7 Hz); 112.5 (d, *J* = 22 Hz); 111.6 (d, *J* = 3.4 Hz); 40.0; 35.0; 33.0; 24.6; 24.1.

Preparation of MRL-871 probe

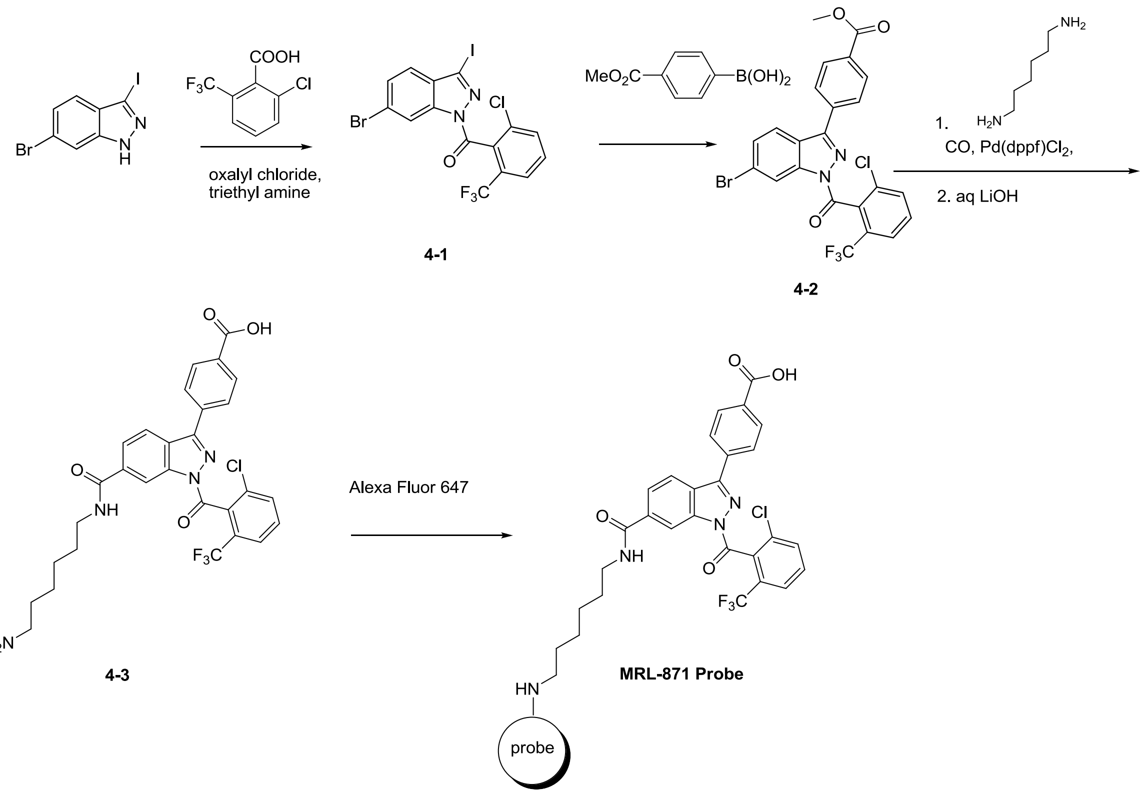

Step 1. Preparation of (6-bromo-3-iodo-1*H*-indazol-1-yl)(2-chloro-6- (trifluoromethyl)phenyl) methanone (4-1). Into a 100-mL 4-necked round-bottom flask purged and maintained with an inert atmosphere of nitrogen was placed a solution of $(COCl)_2$ (56 g) in tetrahydrofuran (560 mL). This was followed by the addition of N,N-dimethylformamide (2.8 mL) dropwise with stirring at room temperature over 20 min. The resulting solution was stirred for 30 min at room temperature, followed by addition of a solution of 2-chloro-6-(trifluoromethyl)benzoic acid (90 g, 1.00 equiv) in tetrahydrofuran (450 mL) dropwise with stirring at room temperature over 20 min. The resulting solution was stirred for 30 min at room temperature. Then it was concentrated under vacuum. The residue was diluted with dichloromethane (1300 mL), then added 6-bromo-3-iodo-1H-indazole (129.4 g, 1.00 equiv). To the mixture was added 4-dimethylaminopyridine (49 g, 1.00 equiv) in portions at room temperature over 30 min. To the mixture was added triethylamine (81g, 2.00 equiv) dropwise with stirring at room temperature over 30 min. The resulting solution was stirred for 4 h at room temperature, then quenched by the addition of 2 L of water/ice. The resulting solution was extracted with dichloromethane. The organic layers were combined, washed with brine, dried and concentrated. The residue was applied onto a silica gel column and eluted with tetrahydrofuran/DCM/hexanes (1:1:20 to afford 170 g (80%) of 6-bromo-1-[[2-chloro-6-(trifluoromethyl)phenyl]carbonyl]-3-iodo-1*H*-indazole as a yellow solid.

Step 2. Preparation methyl 4-(6-bromo-1-(2-chloro-6-(trifluoromethyl) benzoyl)-1*H*-indazol-3-yl)benzoate (4-2). Into a 5-mL 4-necked round-bottom flask purged and maintained with an inert atmosphere of nitrogen was placed 6-bromo-1-[[2-chloro-6-(trifluoromethyl) phenyl]carbonyl]-3-iodo-1*H*-indazole (172 g, 324.85 mmol, 1.00 equiv), [4-(methoxycarbonyl)phenyl]boronic acid (62.7 g, 348.40 mmol, 1.04 equiv), 1,4-dioxane (2500 mL), water (500 mL), KOAc (95.6 g, 974.12 mmol, 2.83 equiv), Pd(dppf)Cl_2.CH_2Cl_2 (26.5 g). The

resulting solution was stirred overnight at 90°C in an oil bath. The reaction was then cooled and quenched by the addition of 500 mL of water/ice. The resulting solution was extracted with 3x1500 mL of dichloromethane. The organic layers were combined, washed with 2x1500 mL of brine, dried over anhydrous sodium sulfate and concentrated under vacuum. The residue was applied onto a silica gel column and eluted with dichloromethane/petroleum ether (1:5). The crude product was re-crystallized from tetrahydrofuran/hexanes in the ratio of 1:5 to afford 80 g (46%) of methyl 4-(6-bromo-1-[[2-chloro-6-(trifluoromethyl) phenyl]carbonyl]-1*H*-indazol-3-yl)benzoate as a white solid. LCMS: 539 $[M+H]^{+}$. 1HNMR (300MHz, CDCl3): δ 7.55-7.71 (4H, m), 7.81-7.85 (3H, m), 8.08-8.12 (2H, m), 8.87-8.88 (1H, d).

Step 3. Preparation of 4-(6-((6-aminohexyl)carbamoyl)-1-(2-chloro-6- (trifluoromethyl)benzoyl)-1*H*-indazol-3-yl)benzoic acid (4-3). To a mixture of methyl 4-(6-bromo-1-(2-chloro-6-(trifluoromethyl) benzoyl)-1*H*-in dazol-3-yl)benzoate (500 mg, 0.930 mmol) and hexane-1,6-diamine (324 mg, 2.79 mmol) in DMF (10.0 mL) were added Pd(dppf)Cl_2 (152 mg, 0.186 mmol) and Et_3N (0.389 ml, 2.79 mmol) under N_2. The suspension was degassed under vacuum and purged with CO several times. The mixture was stirred under CO (50 psi) at 80 °C for 18 hours. LC-MS showed the reaction was finished. The suspension was filtered through a pad of Celite and the filter cake was washed with DCM (20 mL × 3). The combined filtrates were concentrated. The resultant crude residue (~500mg) was dissolved in THF (5.0 mL), followed by the addition of a solution of LiOH (50 mg, 2.088 mmol) in H_2O (5.0 mL). The reaction mixture was stirred at rt for 2 hours. The reaction mixture was concentrated in vacuo and the residue was purified by prep. HPLC (TFA) to afford 4-(6-((6-aminohexyl)carbamoyl) -1-(2-chloro-6-(trifluoromethyl)benzoyl)-1*H*-indazol-3-yl)benzoic acid (60 mg, 0.10 mmol) as a white solid. LCMS: $[M+H]^{+}$: 587.1, found: 587.1. ^{1}H NMR (400MHz, METHANOL-d_4) δ 9.05 (s, 1H), 8.27 (d, *J* = 8.6 Hz, 1H), 8.15 (d, *J* = 8.2 Hz, 2H), 8.03 (d, *J* = 8.2 Hz, 1H), 7.96 (d, *J* = 8.2 Hz, 2H), 7.87 (dd, *J* = 2.9, 7.6 Hz, 2H), 7.81-7.76 (m, 1H), 3.48 (t, *J* = 6.8 Hz, 2H), 2.95 (t, *J* = 7.4 Hz, 2H), 1.72 (d, *J* = 5.9 Hz, 4H), 1.50 (br. s., 4H).

Step 4. Preparation of MRL-871 probe (with Alexa® 647). To a solution of 4-(6-((6-aminohexyl)carbamoyl)-1-(2-chloro-6-(trifluoro methyl) benzoyl)-1*H*-indazol-3-yl)benzoic acid (3.66 mg, 6.24 µmol) in DMSO (0.4ml) at rt was added a solution of Alexa Fluor®647 NHS ester (Succinimidyl Ester, from Invitrogen) (5mg, 4.80 µmol) in DMSO (0.4ml) and Hunig's Base (0.042 mL, 0.24 mmol). The reaction mixture was stirred at rt for 14 h, concentrated and purified by reverse HPLC [UV at 254 nm; column: Waters XBridge 19 x 150 mm, mobile Phase gradient of 0.1% NH_4OH in H_2O/CH_3CN (from 5 to 95%)]to the give the desired product. The structure of this material was confirmed by NMR indicating the presence of both Alex Fluor 647 and **MRL-871** moieties.

Reference

[1] Karstens, W. F. J. *et al.* Rorgammat Inhibitors. WO 2012/106995 (2012).
